# Supplementary material for: Unlocking the mechanism of action: a cost-effective flow cytometry approach for accelerating antimicrobial drug development
Source: Microbiol Spectr. 2024 Mar 14;12(4):e03931-23. doi: 10.1128/spectrum.03931-23 (PMC10986550; doi:10.1128/spectrum.03931-23)
Supplement: Supplemental material — Supplemental figures and tables. [file spectrum.03931-23-s0001.docx]

**Supplementary information for**

**Unlocking the Mechanism of Action: A Cost-Effective Flow Cytometry Approach for Accelerating Antimicrobial Drug Development**

**Authors**

Fabian Mermans^a,b^, Hanna De Baets^a^, Cristina García-Timermans^a^, Wim Teughels^b^, Nico Boon^a#^

**Affiliation**

^a^ Center for Microbial Ecology and Technology, Faculty of Bioscience Engineering, Ghent University, Coupure Links 653, B-9000 Gent, Belgium

^b^ Department of Oral Health Sciences, KU Leuven & Dentistry (Periodontology), University Hospitals Leuven, Kapucijnenvoer 7, B-3000 Leuven, Belgium.

^#^ Correspondence to: Nico Boon, Ghent University, Faculty of Bioscience Engineering; Center of Microbial Ecology and Technology; Coupure Links 653; B-9000 Gent, Belgium: phone: +32 (0)9 264 59 76; fax: +32 (0)9 264 62 48; E-mail: Nico.Boon@UGent.be

**Supplementary 1. Gating of microbial cells**


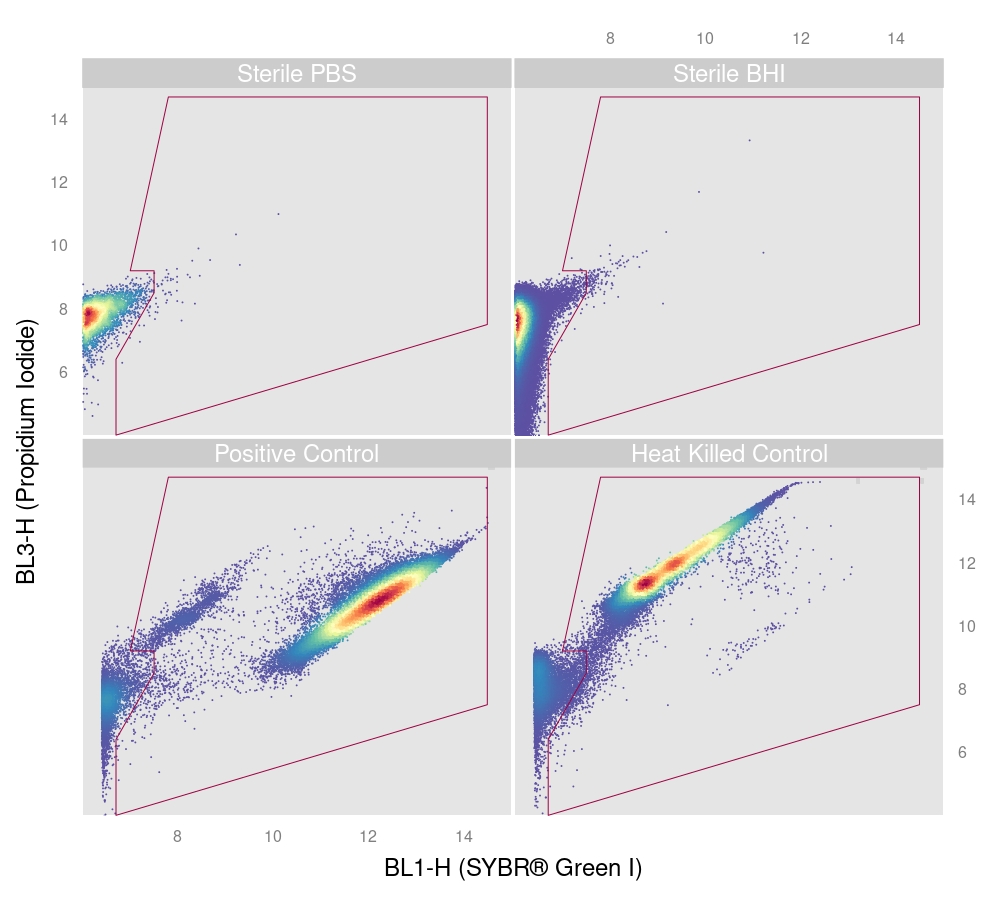


Figure S1. Gating strategy for A. viscosus after 3.5h of treatment with antimicrobials. Negative controls are represented by ‘Sterile PBS’ (dilutant used for microbial cultures), and ‘Sterile BHI’ (growth medium). ‘Positive Control’ depicts an untreated bacterial culture and ‘Heat Killed Control’ shows a heat-treated bacterial culture. The gate is drawn so that background signal is excluded and bacterial cells are included.


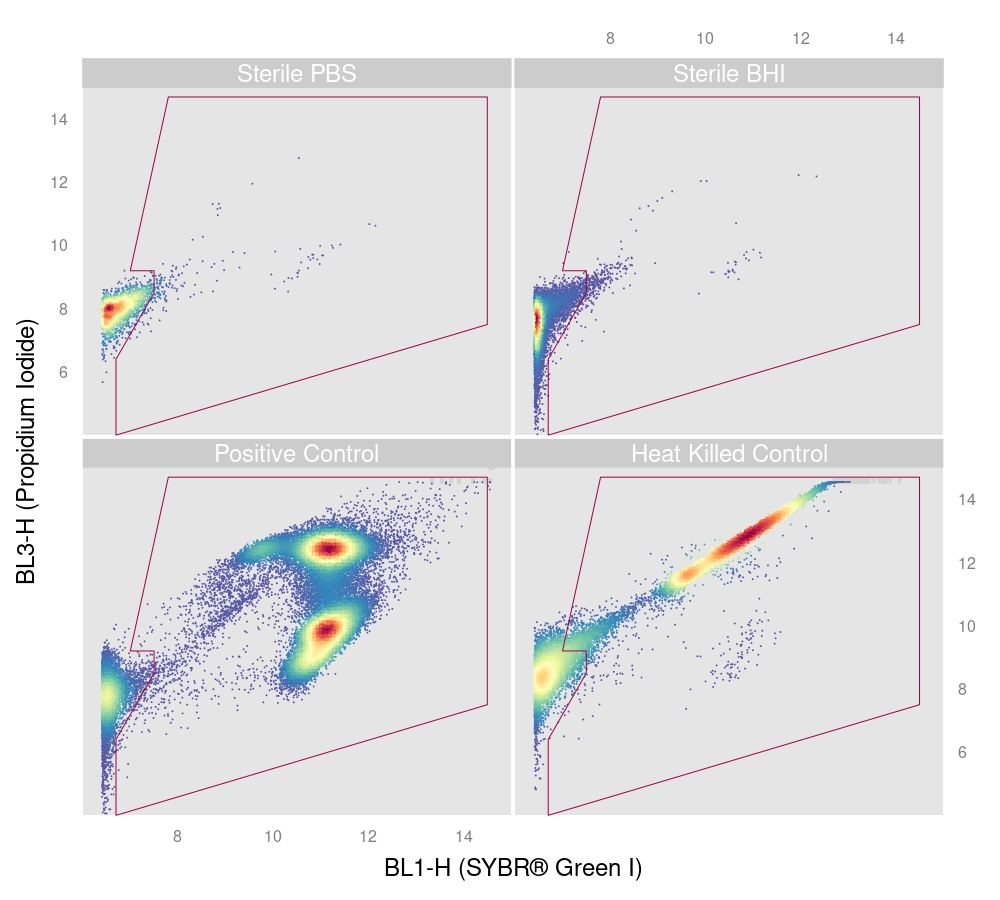


Figure S2. Gating strategy for F. nucleatum after 3.5h of treatment with antimicrobials. Negative controls are represented by ‘Sterile PBS’ (dilutant used for microbial cultures), and ‘Sterile BHI’ (growth medium). ‘Positive Control’ depicts an untreated bacterial culture and ‘Heat Killed Control’ shows a heat-treated bacterial culture. The gate is drawn so that background signal is excluded and bacterial cells are included.


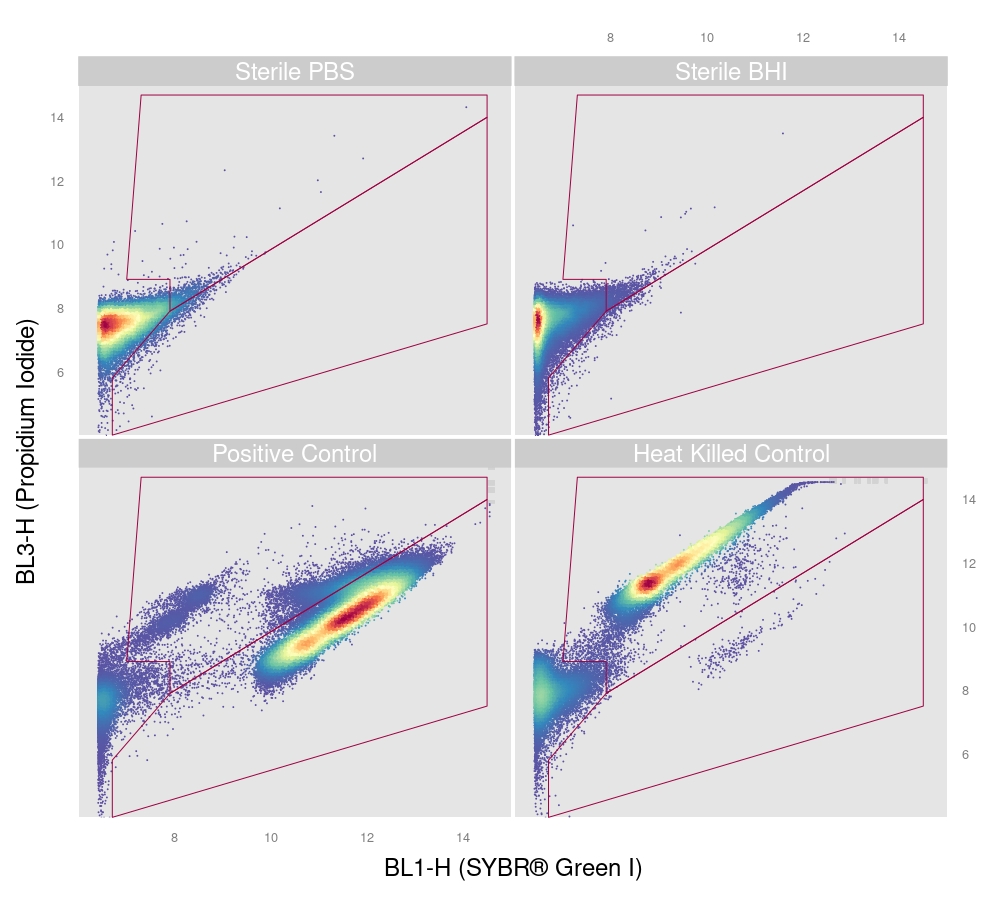


Figure S3. Intact-damaged gating strategy for A. viscosus after 24h of treatment with antimicrobials. Negative controls are represented by ‘Sterile PBS’ (dilutant used for microbial cultures), and ‘Sterile BHI’ (growth medium). ‘Positive Control’ depicts an untreated bacterial culture and ‘Heat Killed Control’ shows a heat-treated bacterial culture. The gates are drawn so that background signal is excluded. The lower gate includes the intact cells that are stained with SYBR® Green I only. The upper gate includes cells that are dual-stained by SGPI and therefore have a damaged membrane.


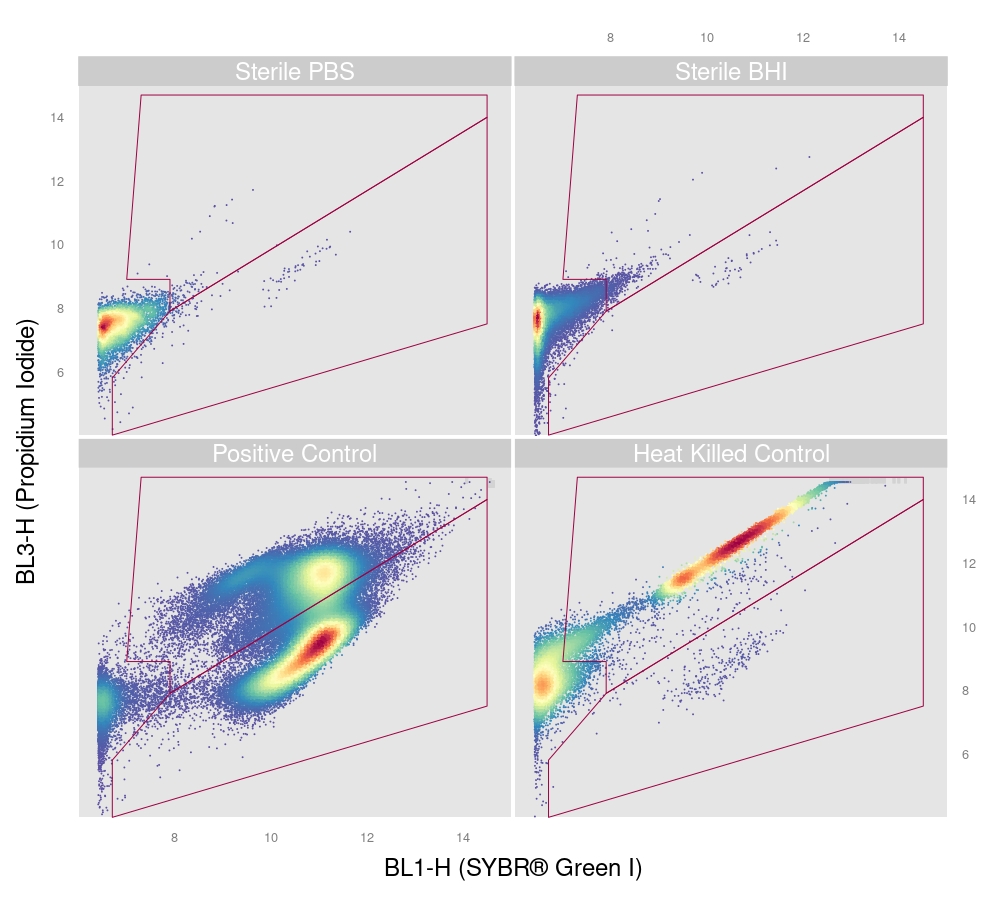


Figure S4. Intact-damaged gating strategy for F. nucleatum after 24h of treatment with antimicrobials. Negative controls are represented by ‘Sterile PBS’ (dilutant used for microbial cultures), and ‘Sterile BHI’ (growth medium). ‘Positive Control’ depicts an untreated bacterial culture and ‘Heat Killed Control’ shows a heat-treated bacterial culture. The gates are drawn so that background signal is excluded. The lower gate includes the intact cells that are stained with SYBR® Green I only. The upper gate includes cells that are dual-stained by SGPI and therefore have a damaged membrane.

**Supplementary 2. Assessment of intact-damaged cell populations**

**
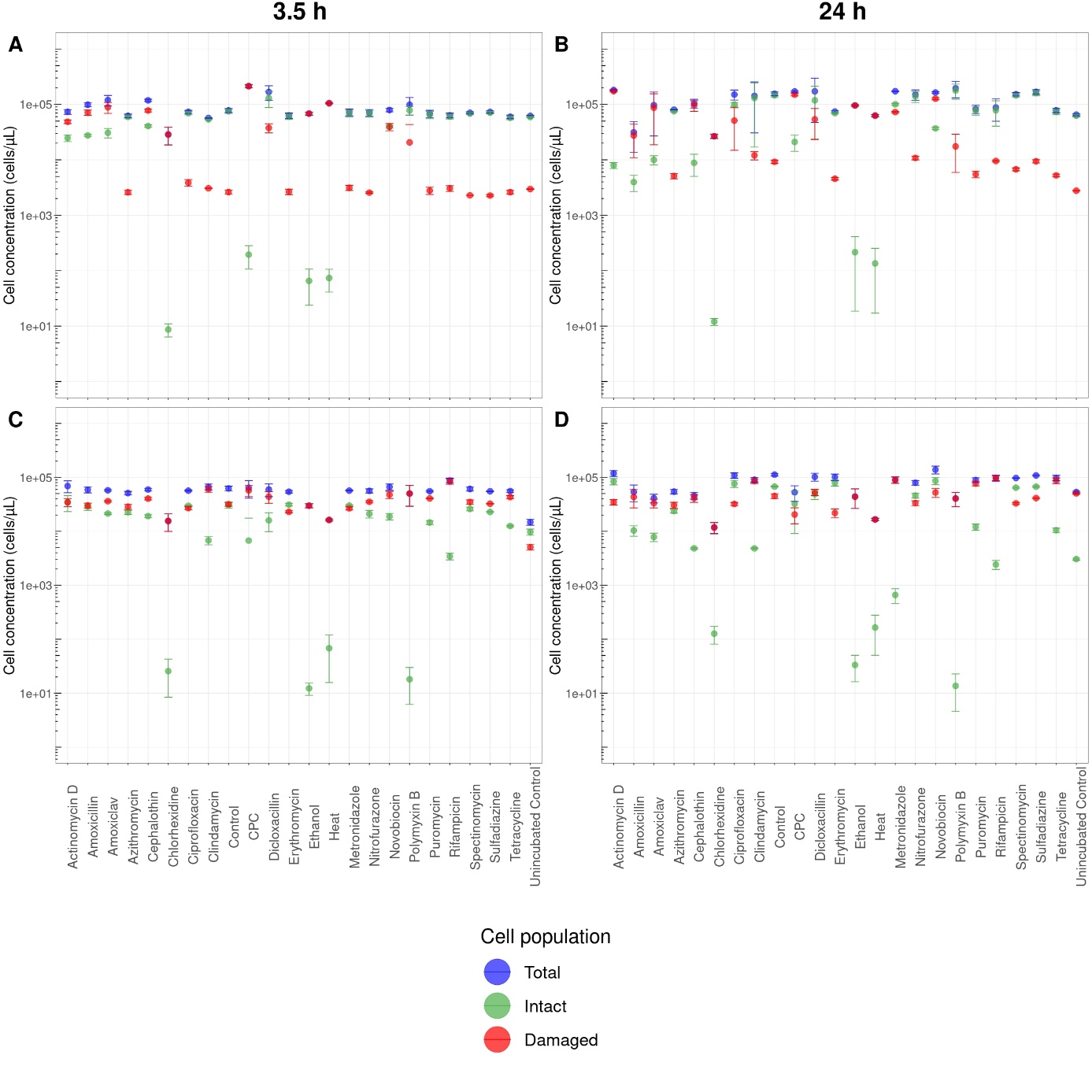
**

Figure S5. Cell concentrations of antimicrobial treated cultures of A. viscosus (A, B) and F. nucleatum (C, D). Concentrations are based on flow cytometric measurement with SGPI staining. Cell populations were differentiated through manual gating of the intact and damaged cell populations.

**
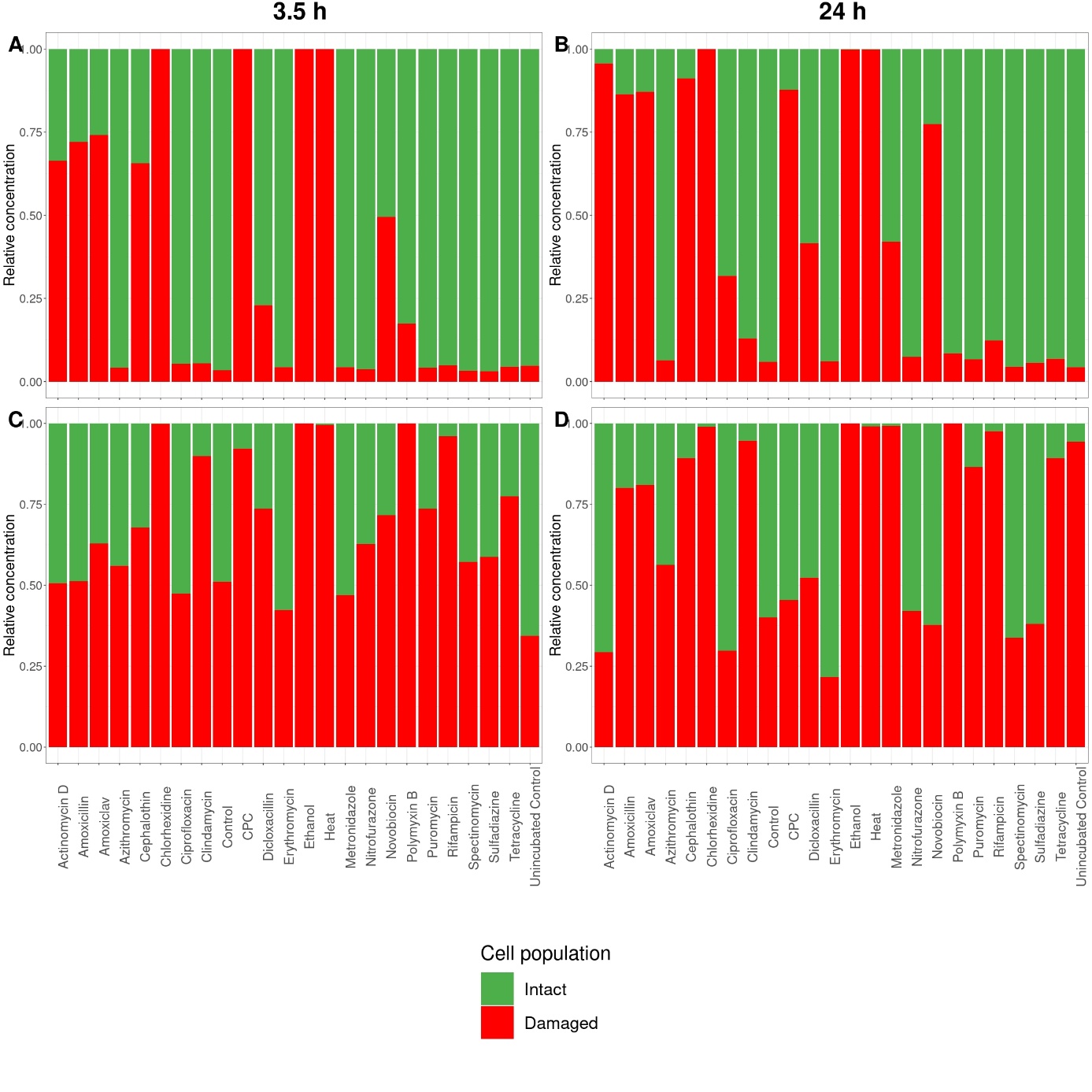
**

Figure S6. Relative abundance of intact and damaged cell populations of antimicrobial treated cultures of A. viscosus (A, B) and F. nucleatum (C, D). Relative abundances are based on flow cytometric measurement with SGPI staining. Cell populations were differentiated through manual gating of the intact and damaged cell populations.

**Supplementary 3. Construction of the phenotypic fingerprints**

Fingerprints were constructed by applying a Gaussian mixture mask to the flow cytometric data. MOA classes were subsampled to an equal number of cells to avoid biased model training towards a specific class. The number of clusters for the Gaussian mixture model (GMM) was determined by optimization of the Bayesian information criterion (BIC) using *PhenoGMM*. Figure S7 shows the BIC for different number of clusters included in the GMM for *A. viscosus* and *F. nucleatum* after 3.5h and 24h of treatment with antimicrobials. Figure S8 shows the BIC for saliva after 24h of treatment with antimicrobials.


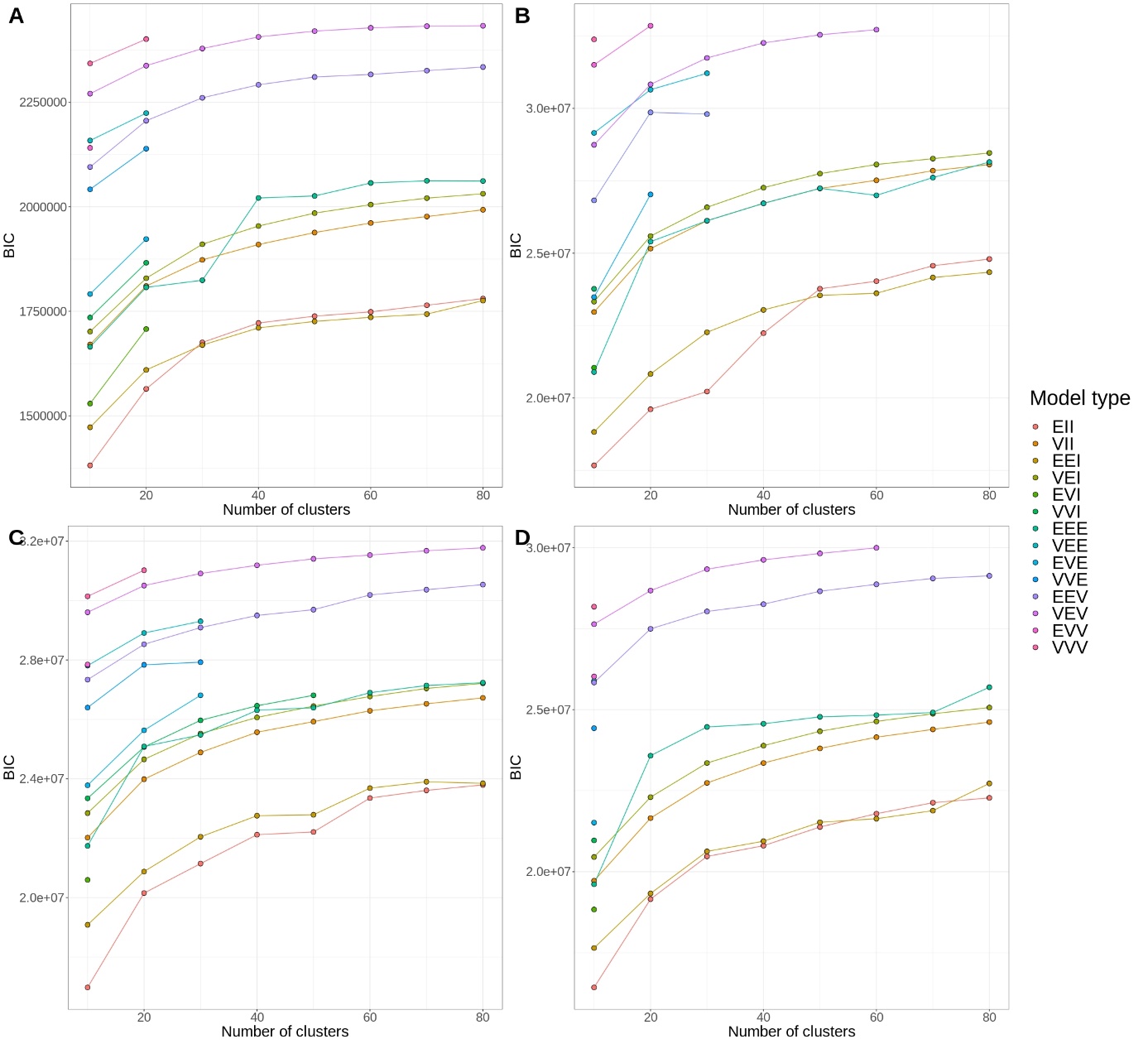


Figure S7. BIC values for different model types for A. viscosus (A, B) and F. nucleatum (C, D) after 3.5h (A, C) and 24h (B, D) of treatment with antimicrobials.

The number of clusters in the final model (i.e. the mask) was decided by determining the number of clusters where the BIC starts to converge to its maximum value for all model types. Using a higher number of clusters would increase computation time with only marginal improvement of the BIC. For *A. viscosus* at both treatment times and for *F. nucleatum* after 3.5h of treatment, the number of clusters was set to 50 in the final model. The number of clusters in the final model was set to 40 for *F. nucleatum* after 24h of treatment. For the saliva, the number of clusters was set to 50.


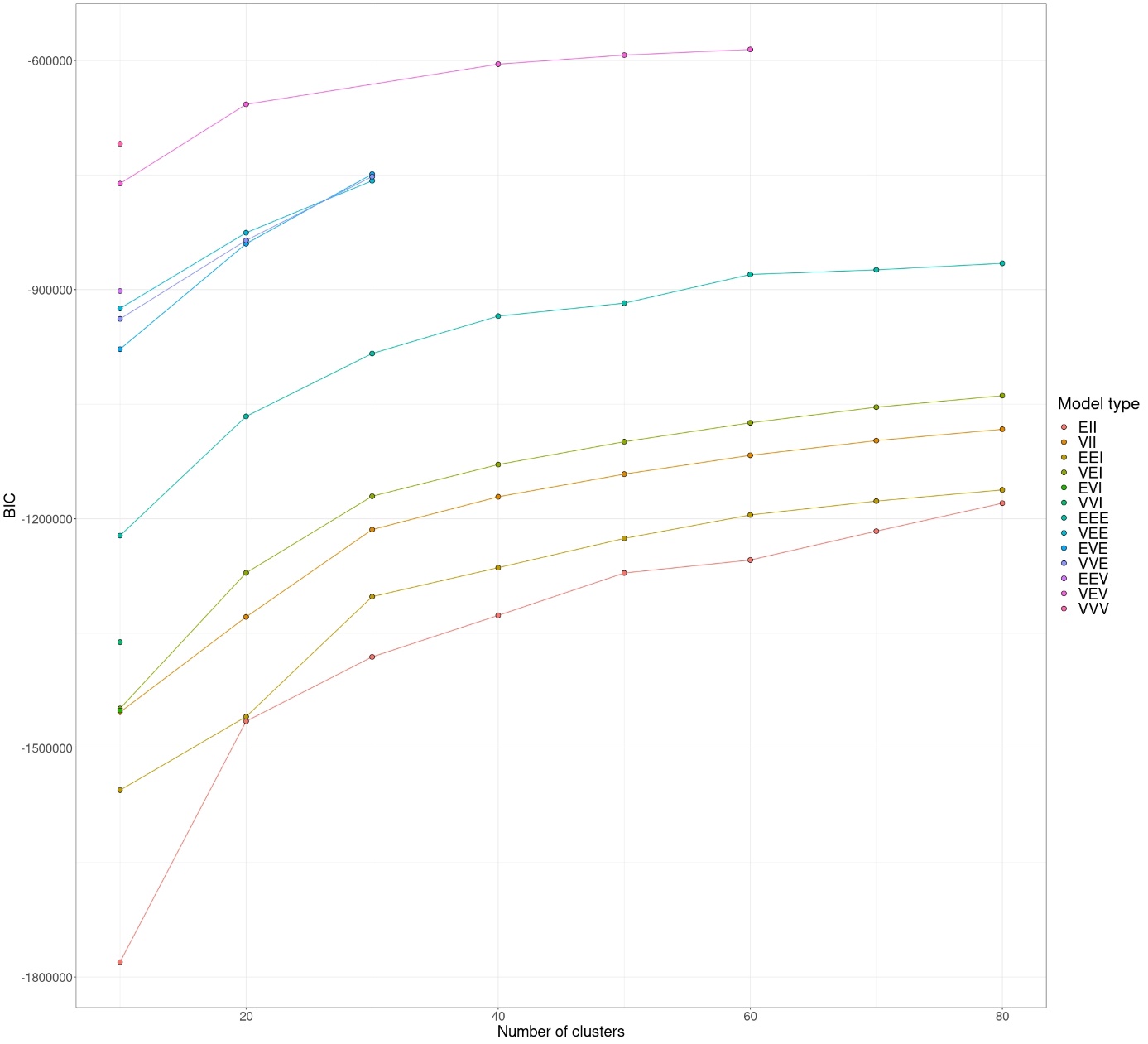


Figure S8. BIC values for different model types for a saliva sample after 24h of treatment with antimicrobials.

Finally, by applying the resulting mask to the data, phenotypic fingerprints could be constructed for each sample. Figure S9 shows a graphical representation of the fingerprints for each sample for *A. viscosus* (A, B) and *F. nucleatum* (C, D). Figure S10 shows a graphical representation of the fingerprints for the saliva samples. In Figure S11 examples of flow cytometry dot plots for *A. viscosus* after 3.5h of treatment are shown, where the color of the dot represents the allocated cluster in the *PhenoGMM* fingerprint. In Figure S12 the same is shown for 24h-treated saliva.


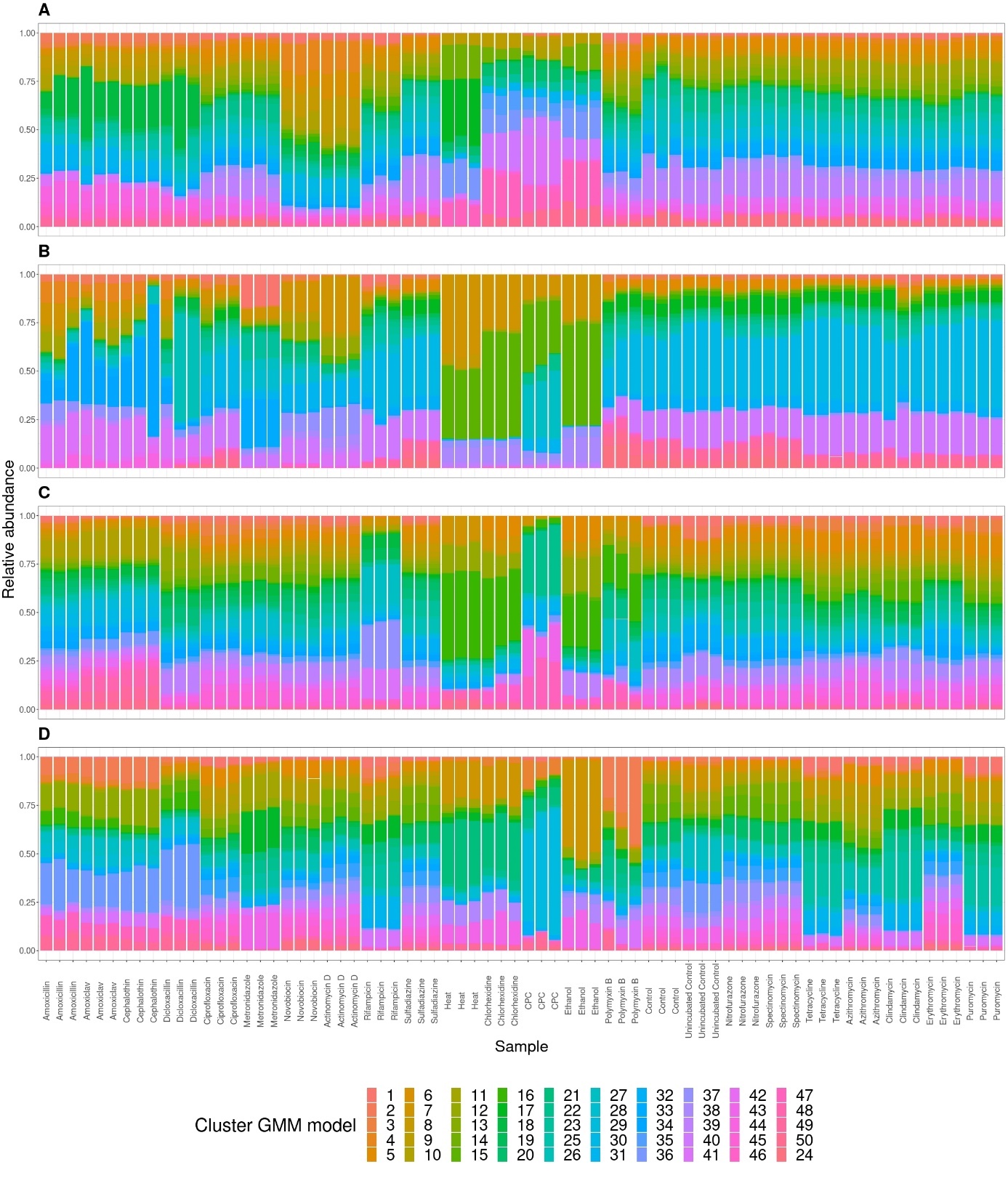


Figure S9. Relative abundance of cells in each cluster of the PhenoGMM generated mask for antimicrobial treated samples of A. viscosus (A, B) and F. nucleatum (C, D) after 3.5h (A, C) and 24h (B, D) of treatment. This representation depicts the phenotypic fingerprint of each sample.


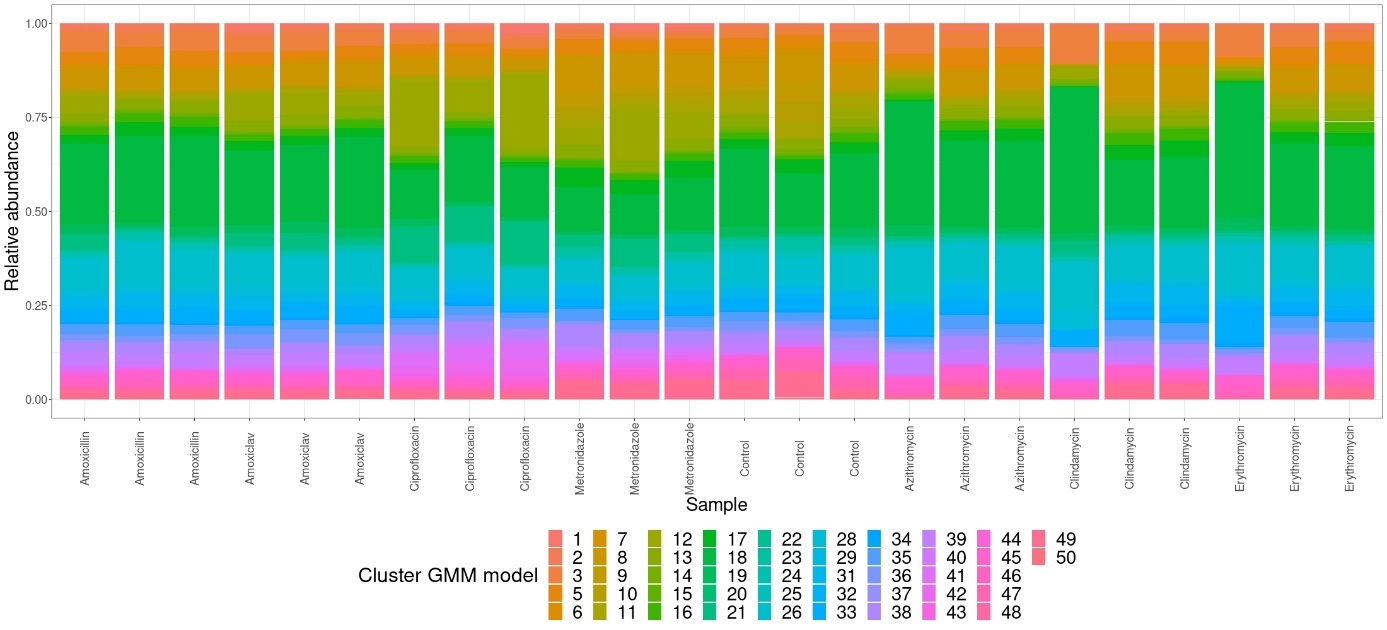


Figure S10. Relative abundance of cells in each cluster of the *PhenoGMM* generated mask for 24h antimicrobial treated saliva. This representation depicts the phenotypic fingerprints of each sample.


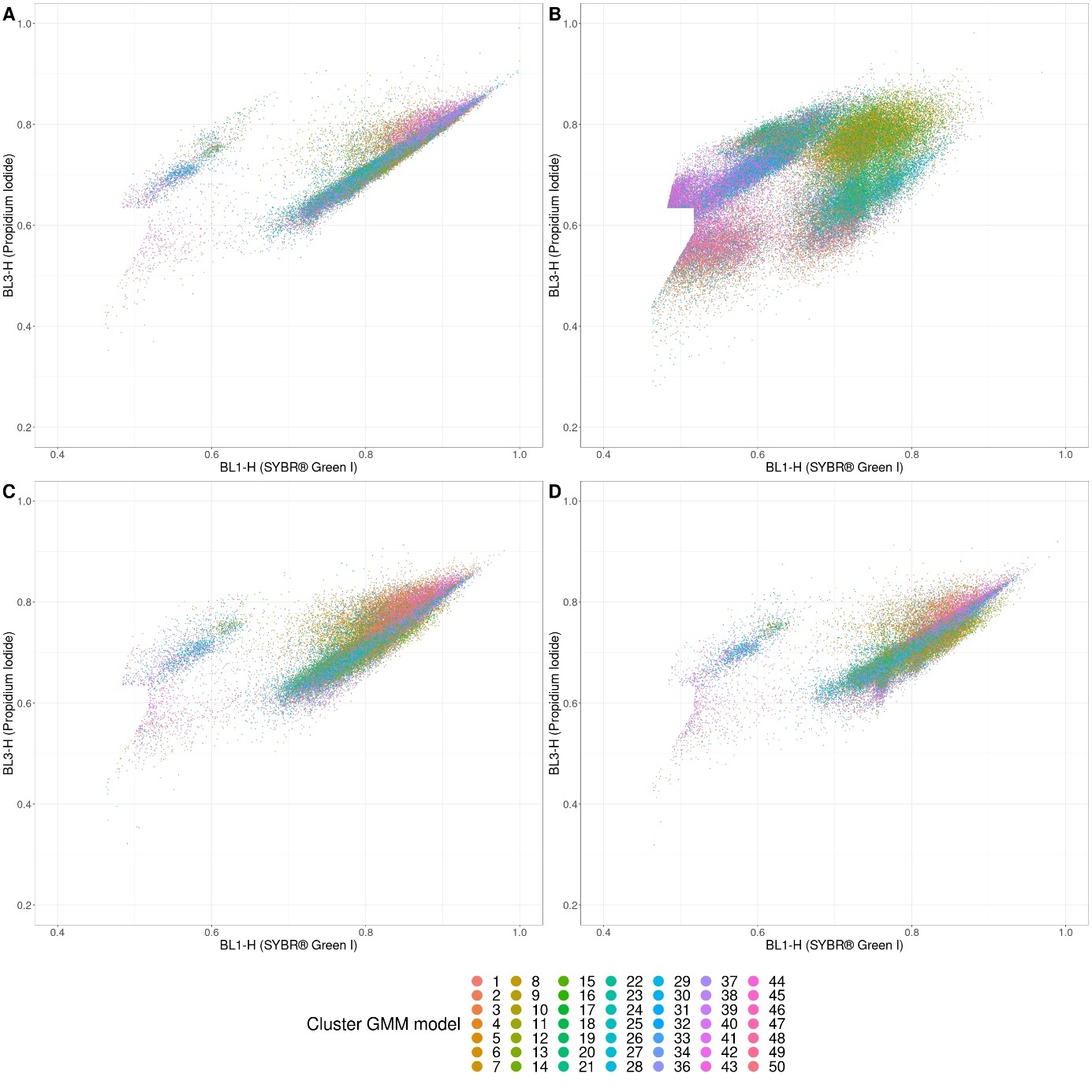


Figure S11. Flow cytometry dot plots of *A. viscosus* after 3.5h of treatment with antimicrobials. Colors indicate the respective cluster from the *PhenoGMM* model to which the specific cells belong. (A) depicts the untreated control, (B) an amoxicillin treated sample belonging to the ‘Cell Wall Synthesis’ class, (C) a ciprofloxacin treated sample belonging to the ‘DNA Replication’ class, and (D) a clindamycin treated sample belonging to the ‘Protein Synthesis – 50S Inhibition’ class.


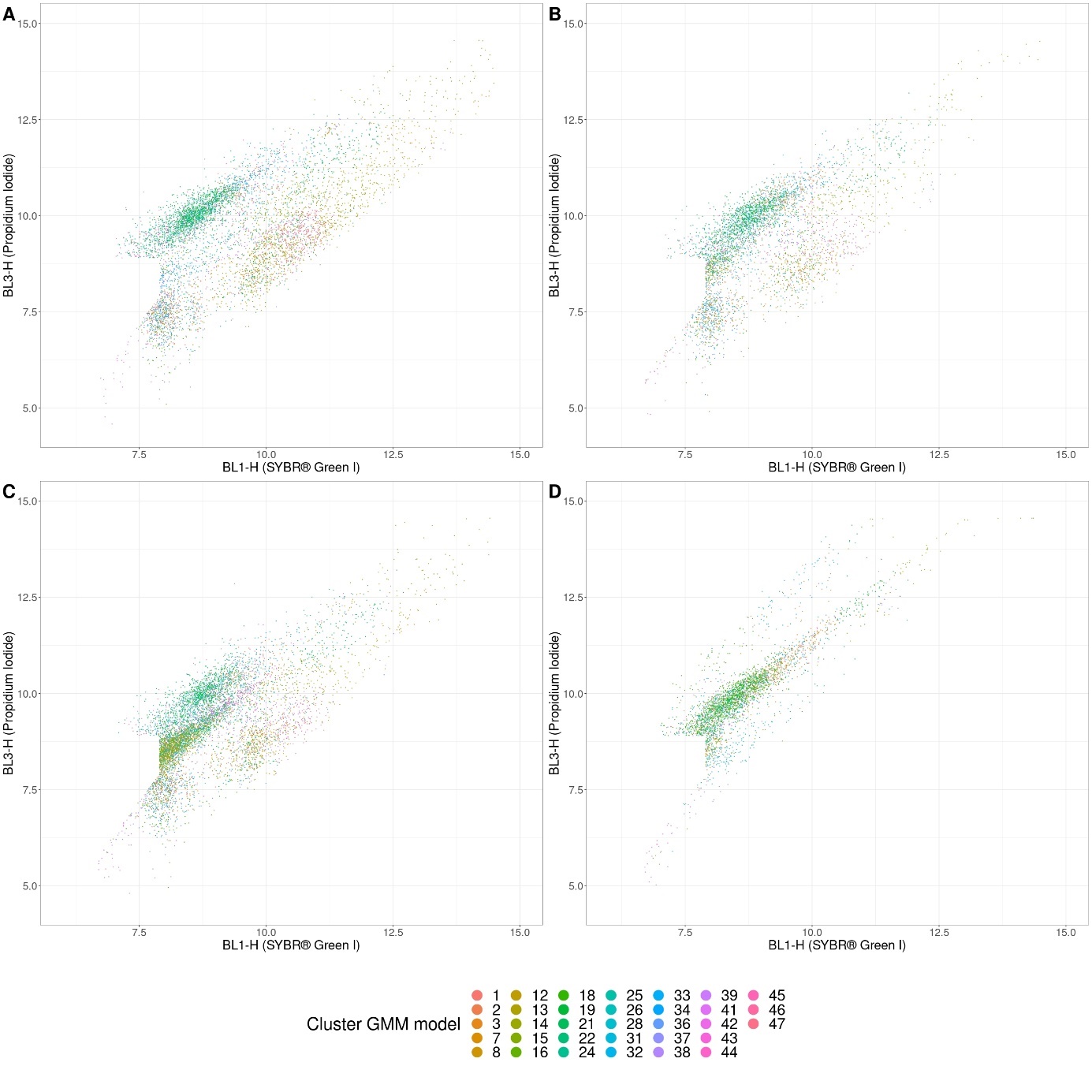


Figure S12. Flow cytometry dot plots of saliva after 24h of treatment with antimicrobials. Colors indicate the respective cluster from the *PhenoGMM* model to which the specific cells belong. (A) depicts the untreated control, (B) an amoxicillin treated sample belonging to the ‘Cell Wall Synthesis’ class, (C) a ciprofloxacin treated sample belonging to the ‘DNA Replication’ class, and (D) a clindamycin treated sample belonging to the ‘Protein Synthesis – 50S Inhibition’ class.

**Supplementary 4. PCoA of phenotypic fingerprints**


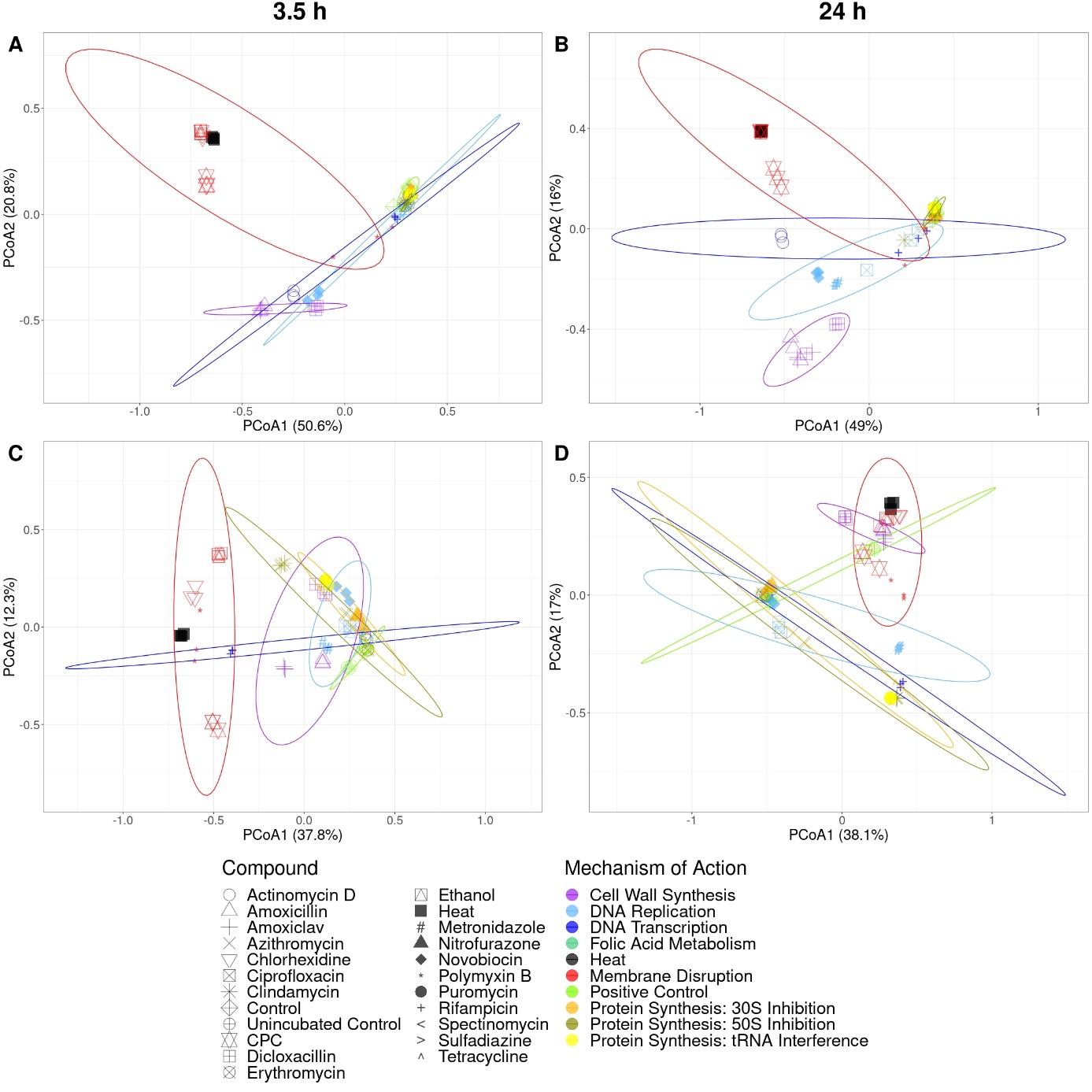


Figure S13. PCoA of flow cytometric fingerprints of A. viscosus (A, B) and F. nucleatum (C, D) after 3.5h (A, C) and 24h (B, D) of treatment with antimicrobials. Fingerprints were generated using PhenoGMM (Phenoflow package). Compounds with the same MOA are grouped by color. Ellipses were drawn at the 95% confidence level. ‘Heat’ indicates the heat-treated control, ‘Control’ indicates the untreated sample that underwent incubation with the antimicrobial treated samples, and ‘Unincubated Control’ indicates the untreated sample that did not undergo consecutive incubation.

**Supplementary 5. NMDS of phenotypic fingerprints of both strains combined**


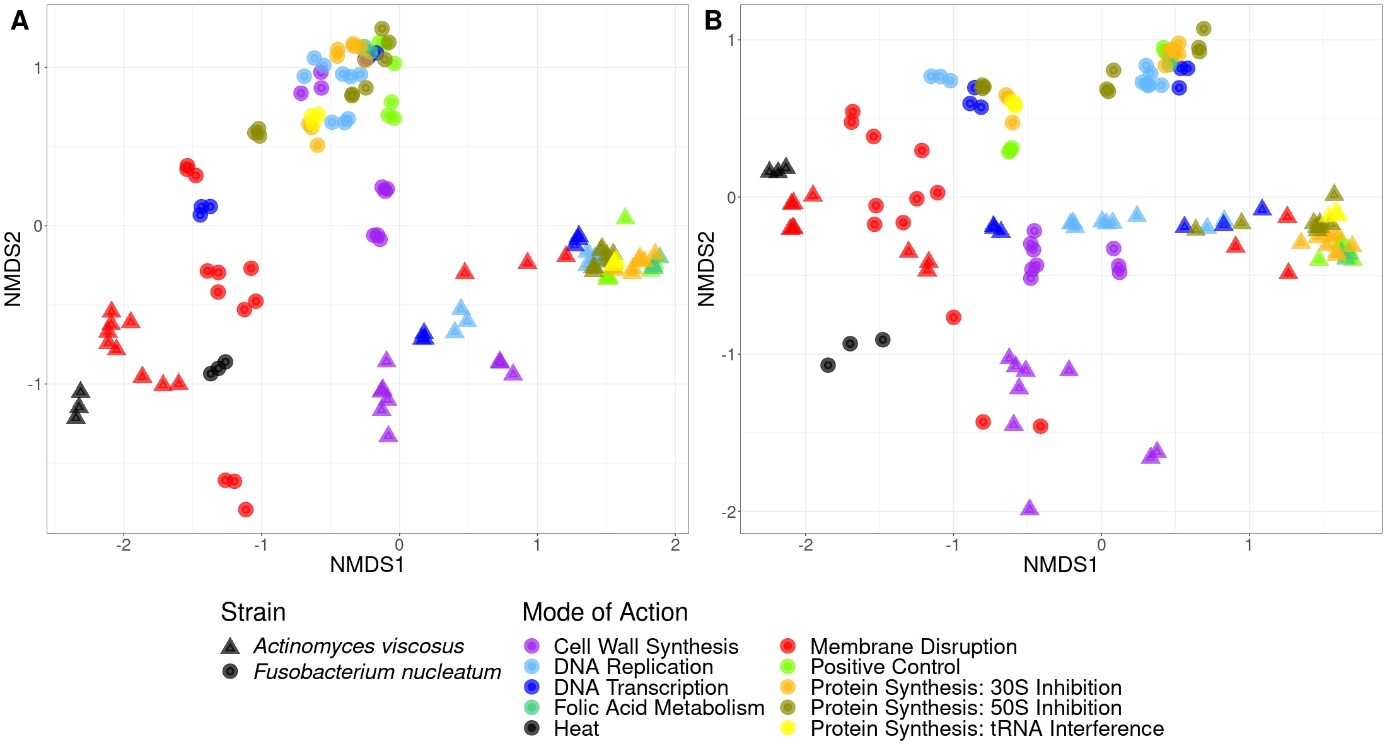


Figure S14. NMDS of flow cytometric fingerprints of A. viscosus and F. nucleatum after 3.5h (A) and 24h (B) of treatment with antimicrobials. Fingerprints were generated using PhenoGMM (Phenoflow package). Compounds with the same MOA are grouped by color. ‘Heat’ indicates to the heat-treated control, ‘Positive Control’ refers to the untreated samples.

**Supplementary 6. Pairwise ANOSIM MOA classes**

Table S1. Pairwise ANOSIM for different MOA classes for A. viscosus and F. nucleatum after 3.5h and 24h of incubation with antimicrobials using PhenoGMM as a fingerprinting algorithm. P-values were adjusted according to the Benjamini and Hochberg method. P-values < 0.01 are indicated with ‘*’, P-values < 0.05 are indicated with ‘.’.

|  | ***A. viscosus*** | | | |  | ***F. nucleatum*** | | | |
| --- | --- | --- | --- | --- | --- | --- | --- | --- | --- |
|  | **3.5h treatment** | | **24h treatment** | |  | **3.5h treatment** | | **24h treatment** | |
| **Comparison** | P adjusted | Significance | P adjusted | Significance |  | P adjusted | Significance | P adjusted | Significance |
| Cell Wall Synthesis-Control | 0.00075 | * | 0.001687 | * |  | 0.003706 | * | 0.001059 | * |
| Cell Wall Synthesis-DNA Replication | 0.000643 | * | 0.001384 | * |  | 0.006428 | * | 0.000818 | * |
| Cell Wall Synthesis-DNA Transcription | 0.001 | * | 0.001687 | * |  | 0.026148 | . | 0.001059 | * |
| Cell Wall Synthesis-Folic Acid Metabolism | 0.008832 | * | 0.008795 | * |  | 0.019712 | . | 0.01309 | . |
| Cell Wall Synthesis-Heat | 0.010285 | . | 0.008999 | * |  | 0.012342 | . | 0.01309 | . |
| Cell Wall Synthesis-Membrane Disruption | 0.001241 | * | 0.001895 | * |  | 0.001 | * | 0.001059 | * |
| Cell Wall Synthesis-Protein Synthesis 30S Inhibition | 0.00075 | * | 0.001 | * |  | 0.003706 | * | 0.0035 | * |
| Cell Wall Synthesis-Protein Synthesis 50S Inhibition | 0.00075 | * | 0.001 | * |  | 0.024574 | . | 0.000818 | * |
| Cell Wall Synthesis-Protein Synthesis tRNA Interference | 0.008279 | * | 0.009195 | * |  | 0.024527 | . | 0.012114 | . |
| Control-DNA Replication | 0.040431 | . | 0.002454 | * |  | 0.001928 | * | 0.155172 | ns |
| Control-DNA Transcription | 0.005785 | * | 0.005275 | * |  | 0.037196 | . | 0.278845 | ns |
| Control-Folic Acid Metabolism | 0.588441 | ns | 0.572568 | ns |  | 0.037196 | . | 0.477414 | ns |
| Control-Heat | 0.018619 | . | 0.019829 | . |  | 0.019839 | . | 0.050263 | ns |
| Control-Membrane Disruption | 0.00075 | * | 0.005275 | * |  | 0.001 | * | 0.01309 | . |
| Control-Protein Synthesis 30S Inhibition | 0.023078 | . | 0.193981 | ns |  | 0.017051 | . | 0.663377 | ns |
| Control-Protein Synthesis 50S Inhibition | 0.001742 | * | 0.005657 | * |  | 0.022858 | . | 0.172268 | ns |
| Control-Protein Synthesis tRNA Interference | 0.341966 | ns | 0.017367 | . |  | 0.020296 | . | 0.050313 | ns |
| DNA Replication-DNA Transcription | 0.040625 | . | 0.138176 | ns |  | 0.03923 | . | 0.155172 | ns |
| DNA Replication-Folic Acid Metabolism | 0.058556 | ns | 0.016713 | . |  | 0.018223 | . | 0.168515 | ns |
| DNA Replication-Heat | 0.008249 | * | 0.009562 | * |  | 0.012342 | . | 0.01309 | . |
| DNA Replication-Membrane Disruption | 0.001969 | * | 0.005999 | * |  | 0.001 | * | 0.001059 | * |
| DNA Replication-Protein Synthesis 30S Inhibition | 0.000643 | * | 0.001 | * |  | 0.019712 | . | 0.050263 | ns |
| DNA Replication-Protein Synthesis 50S Inhibition | 0.00075 | * | 0.001384 | * |  | 0.089726 | ns | 0.207934 | ns |
| DNA Replication-Protein Synthesis tRNA Interference | 0.125464 | ns | 0.01506 | . |  | 0.225965 | ns | 0.324454 | ns |
| DNA Transcription-Folic Acid Metabolism | 0.031698 | . | 0.035178 | . |  | 0.037196 | . | 0.490012 | ns |
| DNA Transcription-Heat | 0.018619 | . | 0.035178 | . |  | 0.036786 | . | 0.050263 | ns |
| DNA Transcription-Membrane Disruption | 0.003176 | * | 0.051681 | ns |  | 0.001636 | * | 0.007105 | * |
| DNA Transcription-Protein Synthesis 30S Inhibition | 0.001742 | * | 0.001895 | * |  | 0.017051 | . | 0.083242 | ns |
| DNA Transcription-Protein Synthesis 50S Inhibition | 0.001241 | * | 0.00375 | * |  | 0.065209 | ns | 0.278845 | ns |
| DNA Transcription-Protein Synthesis tRNA Interference | 0.031698 | . | 0.032161 | . |  | 0.452512 | ns | 0.490012 | ns |
| Folic Acid Metabolism-Heat | 0.109756 | ns | 0.115385 | ns |  | 0.121428 | ns | 0.155172 | ns |
| Folic Acid Metabolism-Membrane Disruption | 0.005108 | * | 0.005999 | * |  | 0.009691 | * | 0.009408 | * |
| Folic Acid Metabolism-Protein Synthesis 30S Inhibition | 0.053245 | ns | 0.636236 | ns |  | 0.164753 | ns | 0.982402 | ns |
| Folic Acid Metabolism-Protein Synthesis 50S Inhibition | 0.009163 | * | 0.015575 | . |  | 0.179299 | ns | 0.451376 | ns |
| Folic Acid Metabolism-Protein Synthesis tRNA Interference | 0.109756 | ns | 0.115385 | ns |  | 0.121428 | ns | 0.155172 | ns |
| Heat-Membrane Disruption | 0.10463 | ns | 0.319259 | ns |  | 0.289453 | ns | 0.618926 | ns |
| Heat-Protein Synthesis 30S Inhibition | 0.008653 | * | 0.007649 | * |  | 0.012102 | . | 0.01309 | . |
| Heat-Protein Synthesis 50S Inhibition | 0.007567 | * | 0.009562 | * |  | 0.012342 | . | 0.013763 | . |
| Heat-Protein Synthesis tRNA Interference | 0.109756 | ns | 0.115385 | ns |  | 0.121428 | ns | 0.155172 | ns |
| Membrane Disruption-Protein Synthesis 30S Inhibition | 0.000643 | * | 0.002454 | * |  | 0.001 | * | 0.000818 | * |
| Membrane Disruption-Protein Synthesis 50S Inhibition | 0.000643 | * | 0.003807 | * |  | 0.001 | * | 0.000818 | * |
| Membrane Disruption-Protein Synthesis tRNA Interference | 0.00586 | * | 0.044727 | . |  | 0.012102 | . | 0.069193 | ns |
| Protein Synthesis 30S Inhibition-Protein Synthesis 50S Inhibition | 0.000643 | * | 0.01664 | . |  | 0.121428 | ns | 0.168515 | ns |
| Protein Synthesis 30S Inhibition-Protein Synthesis tRNA Interference | 0.119131 | ns | 0.056149 | ns |  | 0.263759 | ns | 0.618926 | ns |
| Protein Synthesis 50S Inhibition-Protein Synthesis tRNA Interference | 0.008264 | * | 0.197445 | ns |  | 0.714329 | ns | 0.276625 | ns |

**Supplementary 7. Confusion matrices of random forest classifiers**


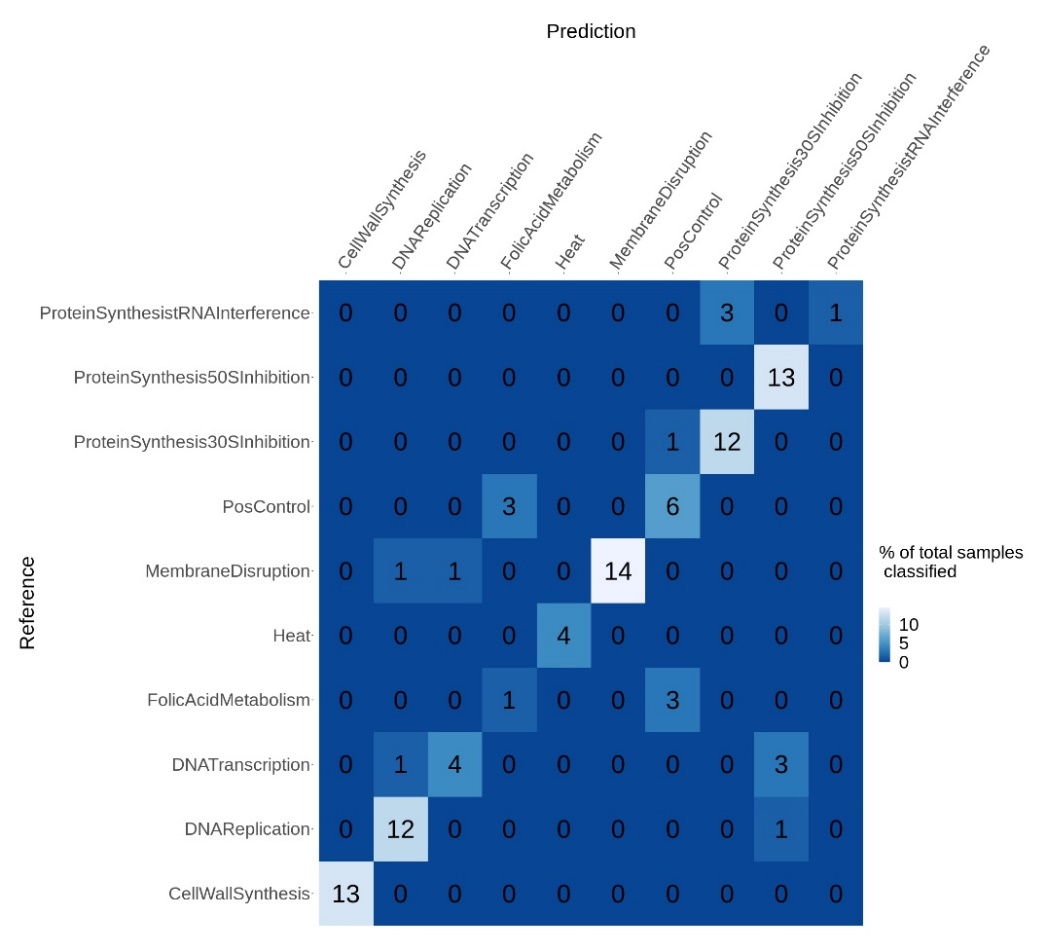


Figure S15. Confusion matrix for random forest model based on A. viscosus after 3.5h of treatment.


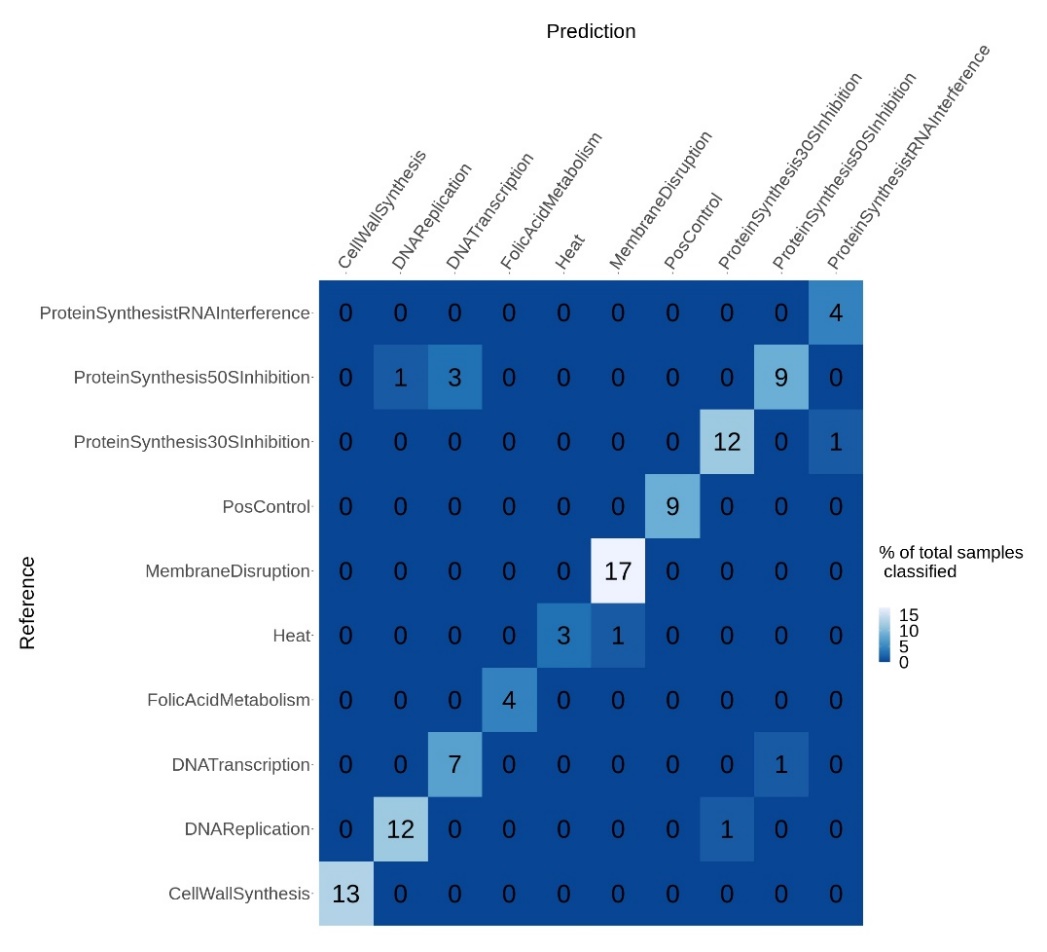


Figure S16. Confusion matrix for random forest model based on F. nucleatum after 3.5h of treatment.

**
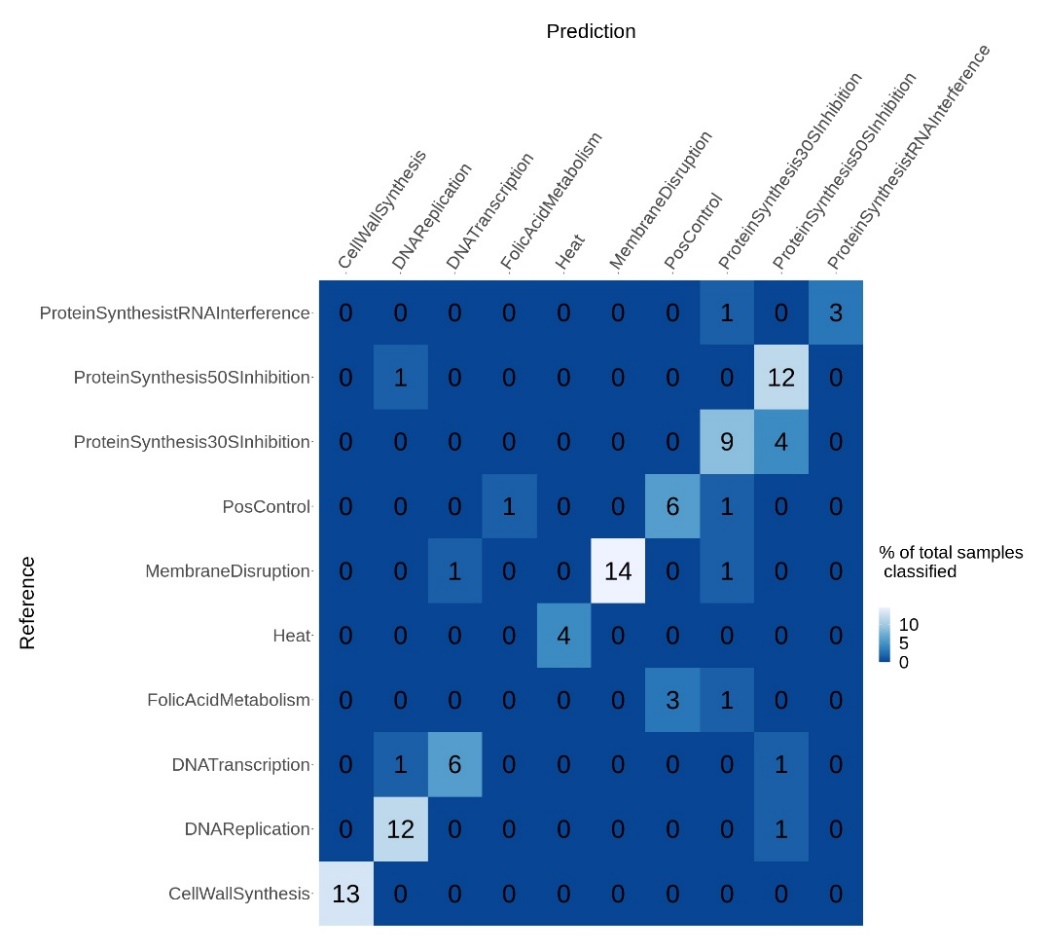
**

Figure S17. Confusion matrix for random forest model based on A. viscosus after 24h of treatment.

**
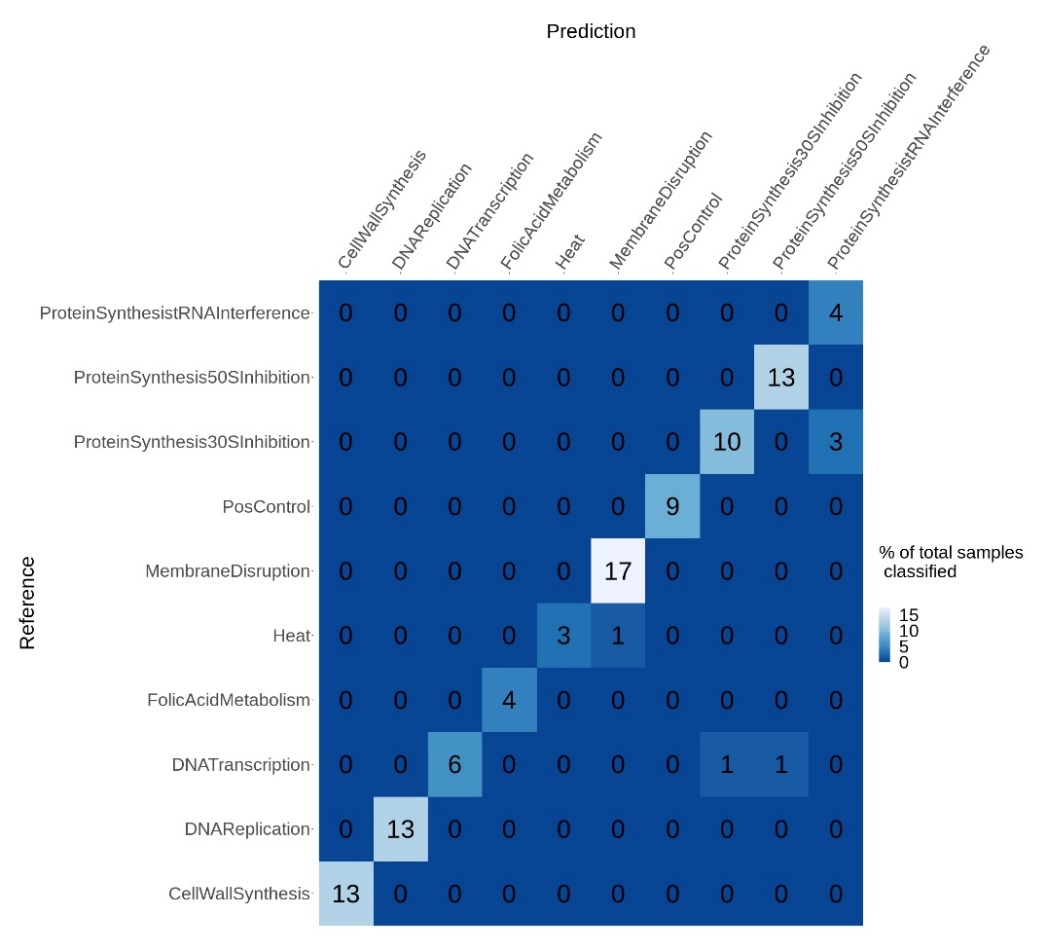
**

Figure S18. Confusion matrix for random forest model based on F. nucleatum after 24h of treatment.

**Supplementary 8. Variable importance for random forest classifiers**


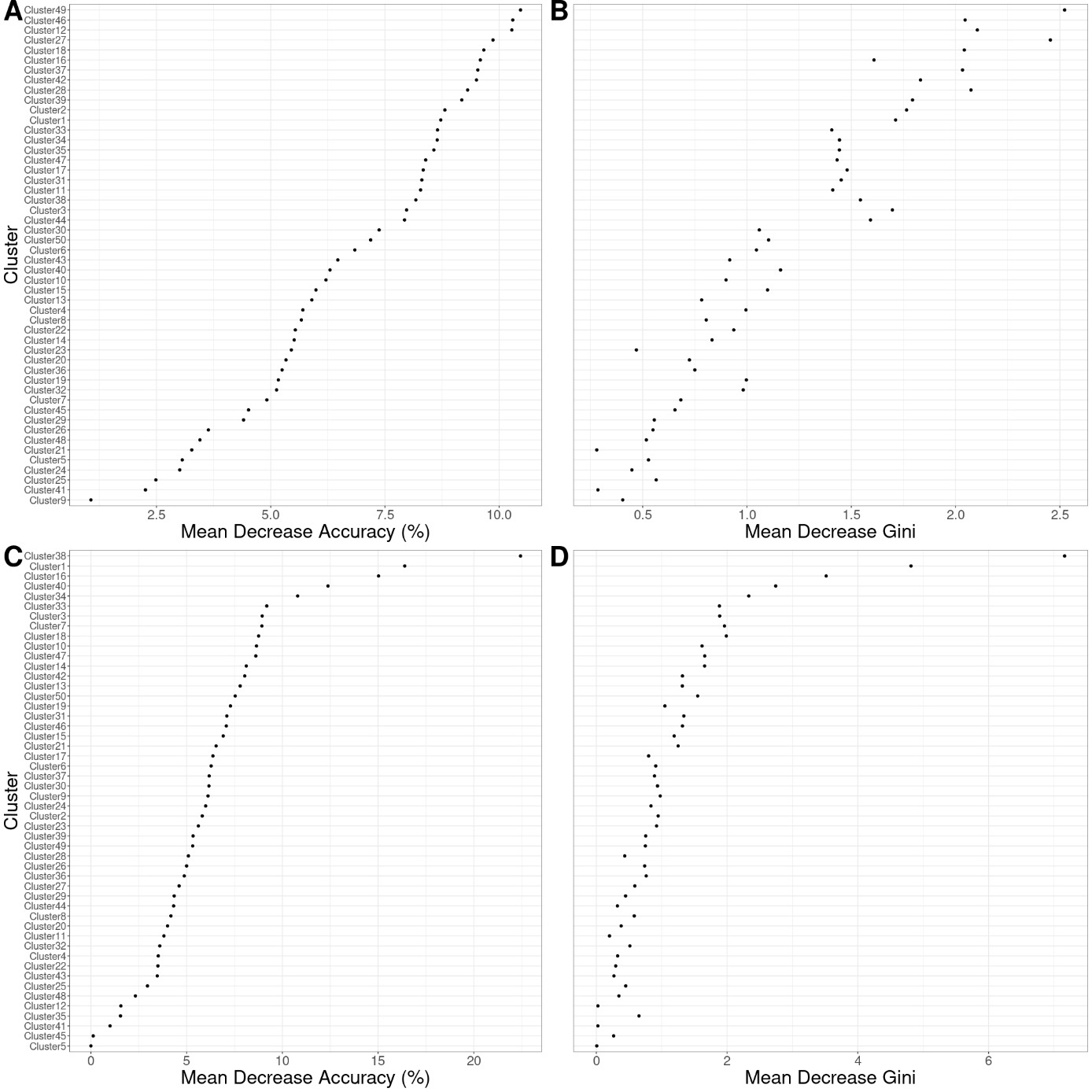


Figure S19. Overall variable importance for *A. viscosus* after 3.5h (A, B) and 24h (C, D) of treatment. (A) and (C) show the mean decrease in accuracy when the respective cluster is excluded from the random forest model. (B) and (D) depict the mean decrease in Gini coefficient when the cluster is excluded from the random forest model. The higher the value of the mean decrease in accuracy or the mean decrease in Gini coefficient, the more important the cluster is in the model.


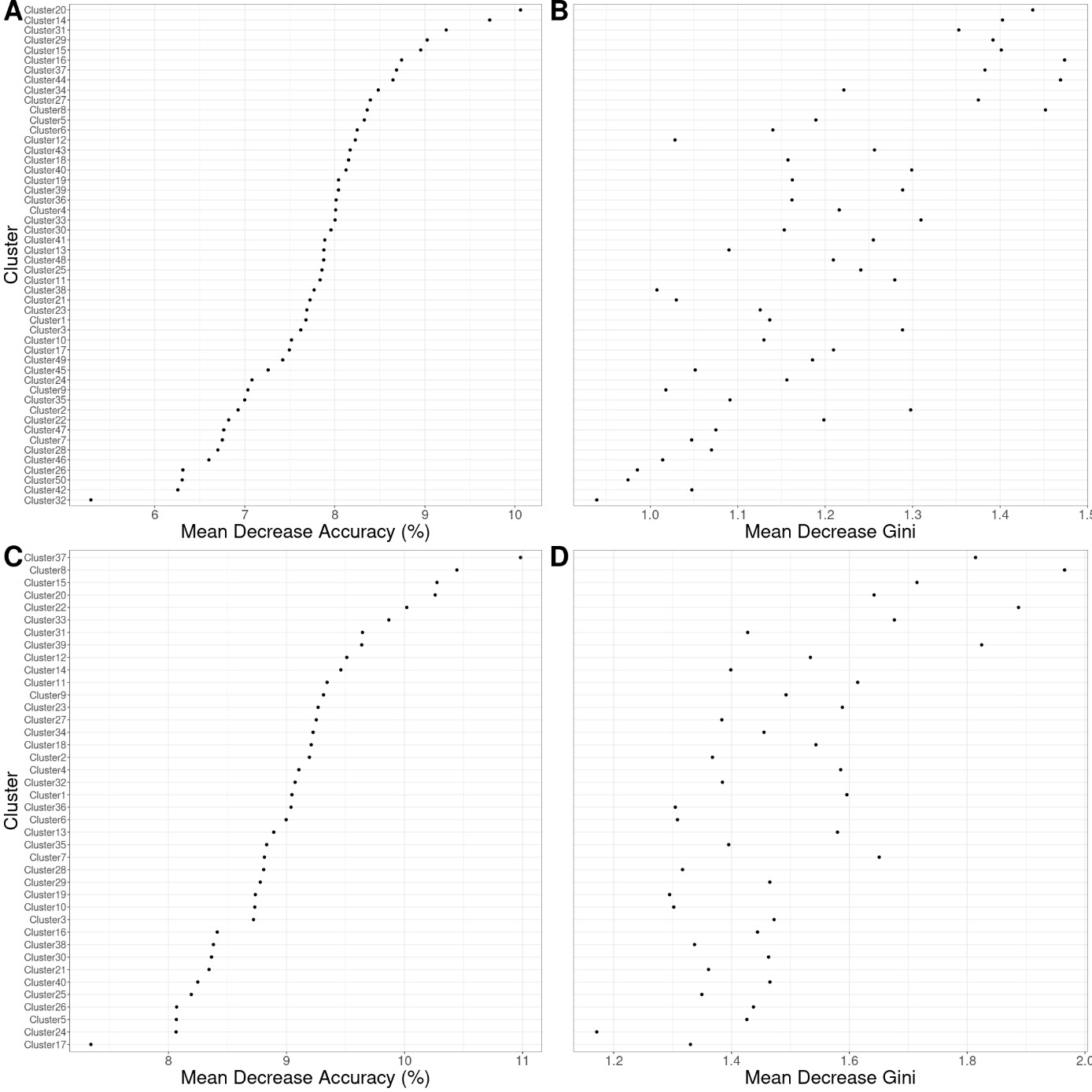


Figure S20. Overall variable importance for *F. nucleatum* after 3.5h (A, B) and 24h (C, D) of treatment. (A) and (C) show the mean decrease in accuracy when the respective cluster is excluded from the random forest model. (B) and (D) depict the mean decrease in Gini coefficient when the cluster is excluded from the random forest model. The higher the value of the mean decrease in accuracy or the mean decrease in Gini coefficient, the more important the cluster is in the model.


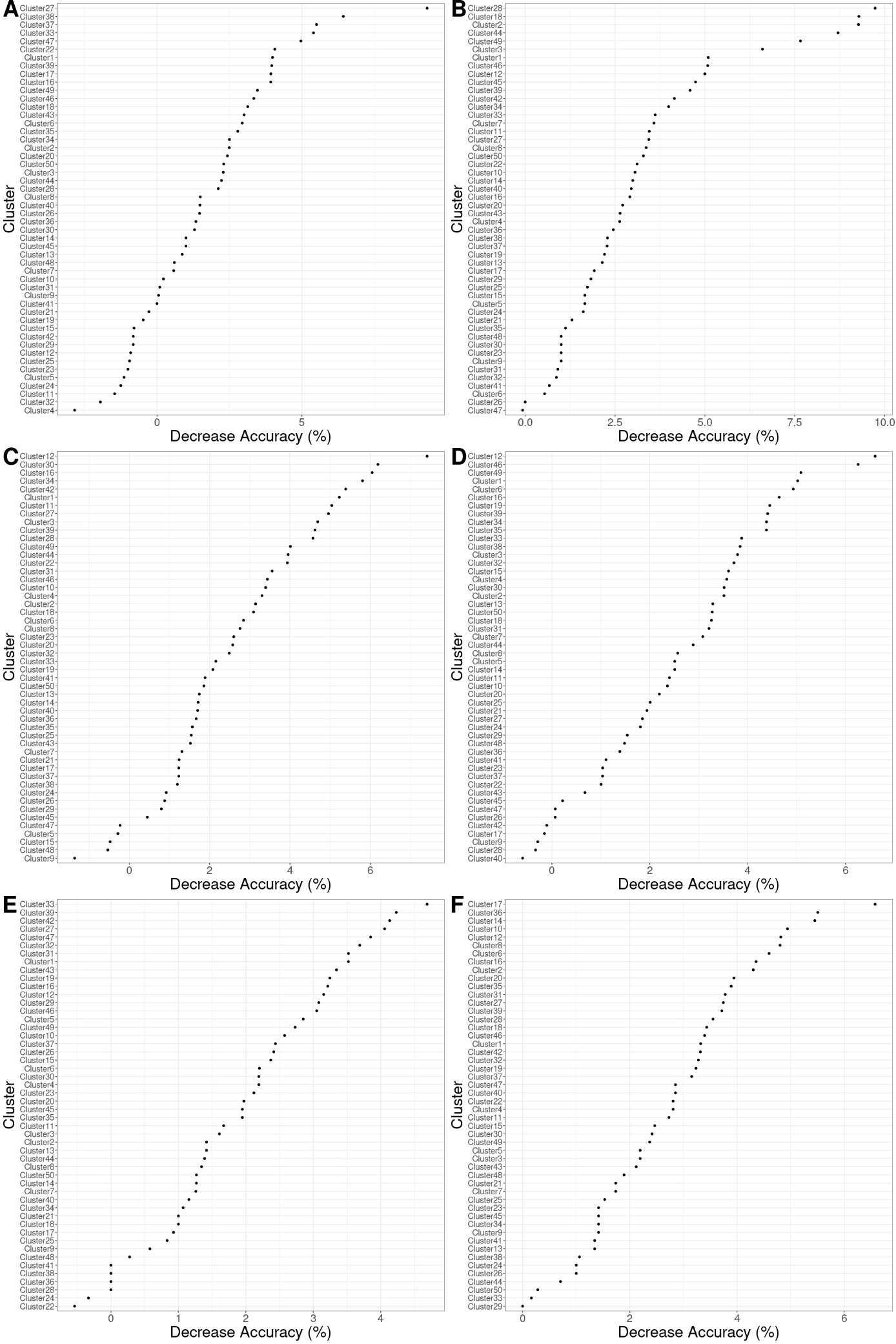


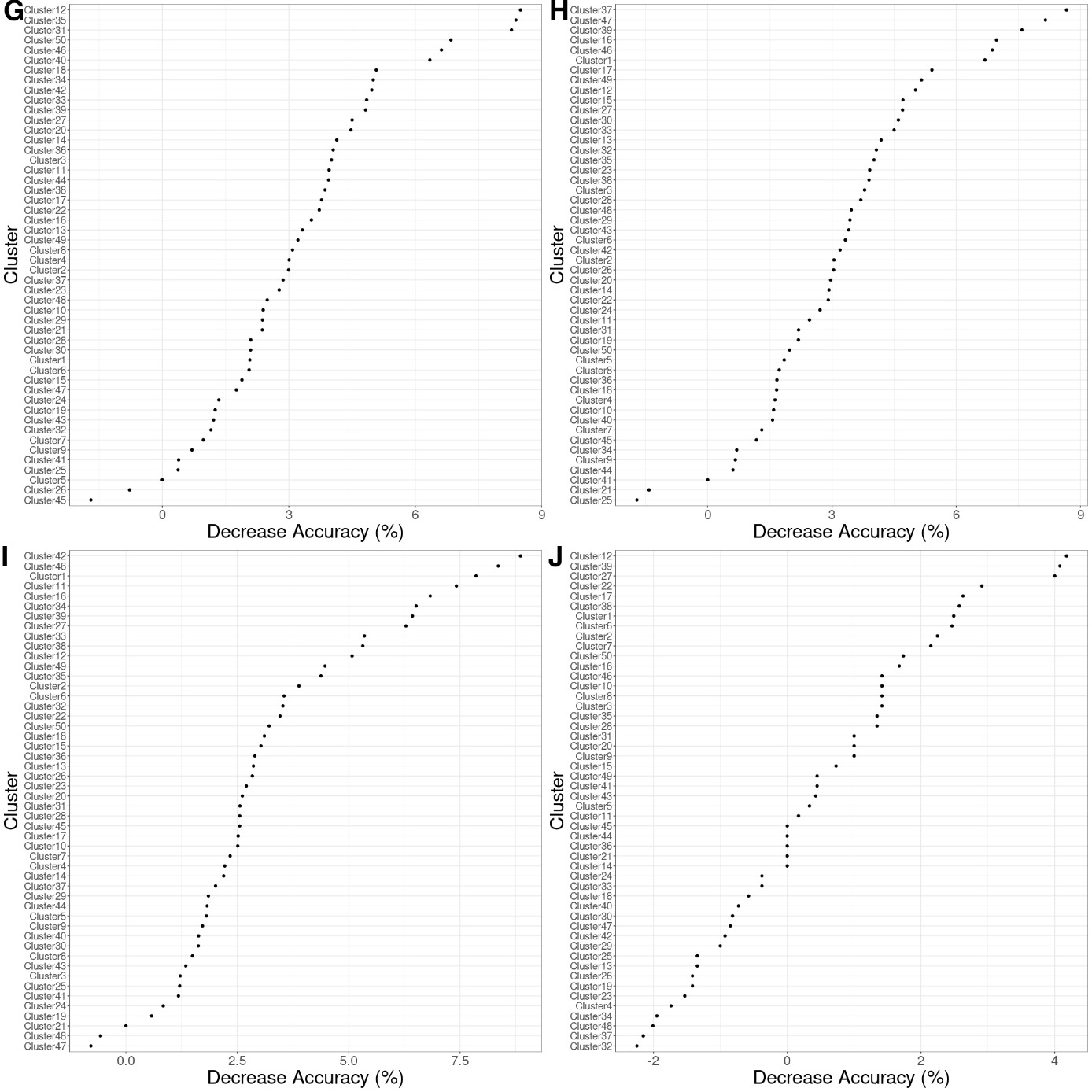


Figure S21. Variable importance per MOA class for *A. viscosus* after 3.5h of treatment. (A) ‘Control’ class, (B) ‘Cell Wall Synthesis’ class, (C) ‘DNA Replication’ class, (D) ‘DNA Transcription’ class, (E) ‘Folic Acid Metabolism’ class, (F) ‘Heat’ class, (G) ‘Membrane Disruption’ class, (H) ‘Protein Synthesis – 30S Inhibition’ class, (I) ‘Protein Synthesis – 50S Inhibition’ class, (J) ‘Protein Synthesis – tRNA Transcription’ class.


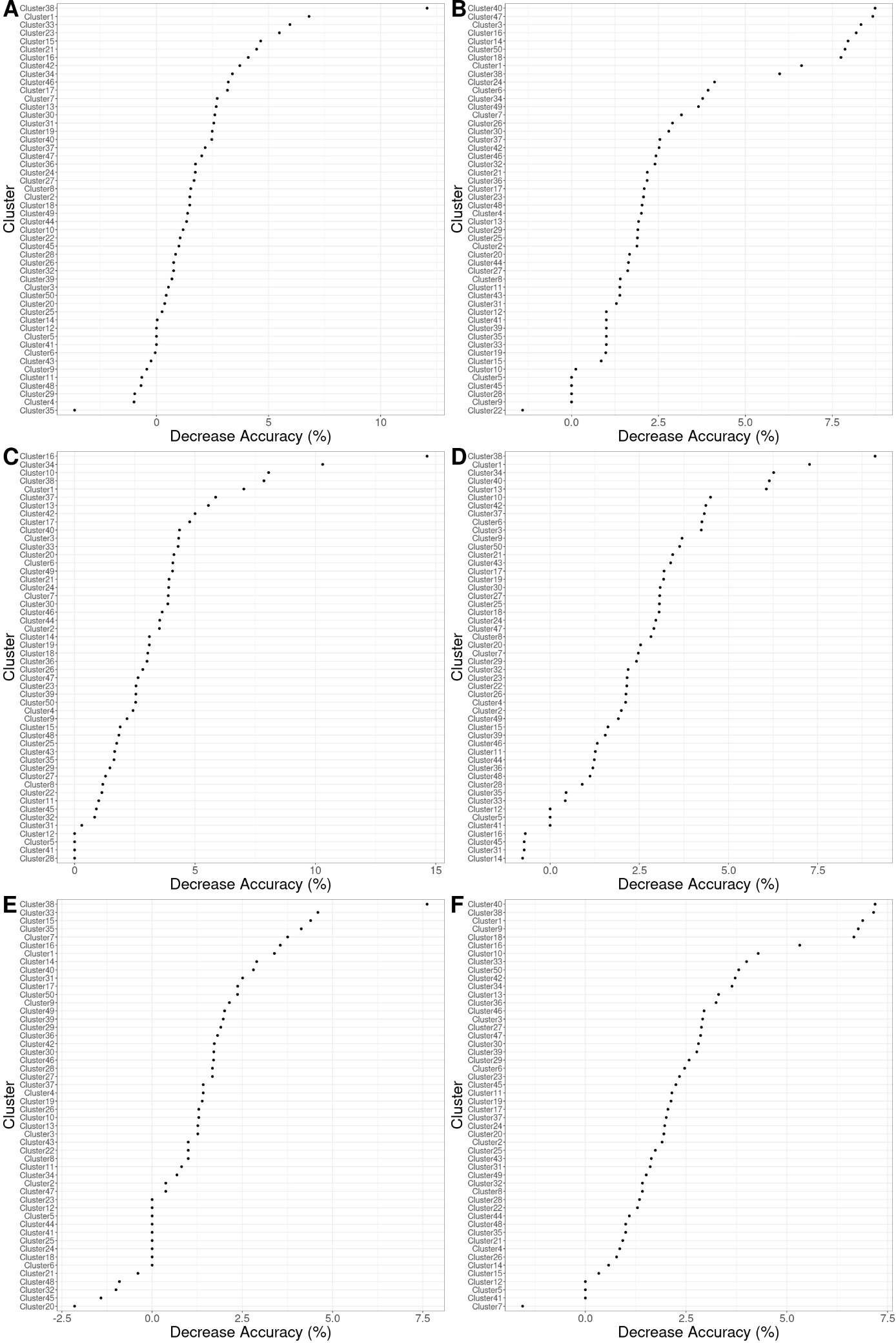


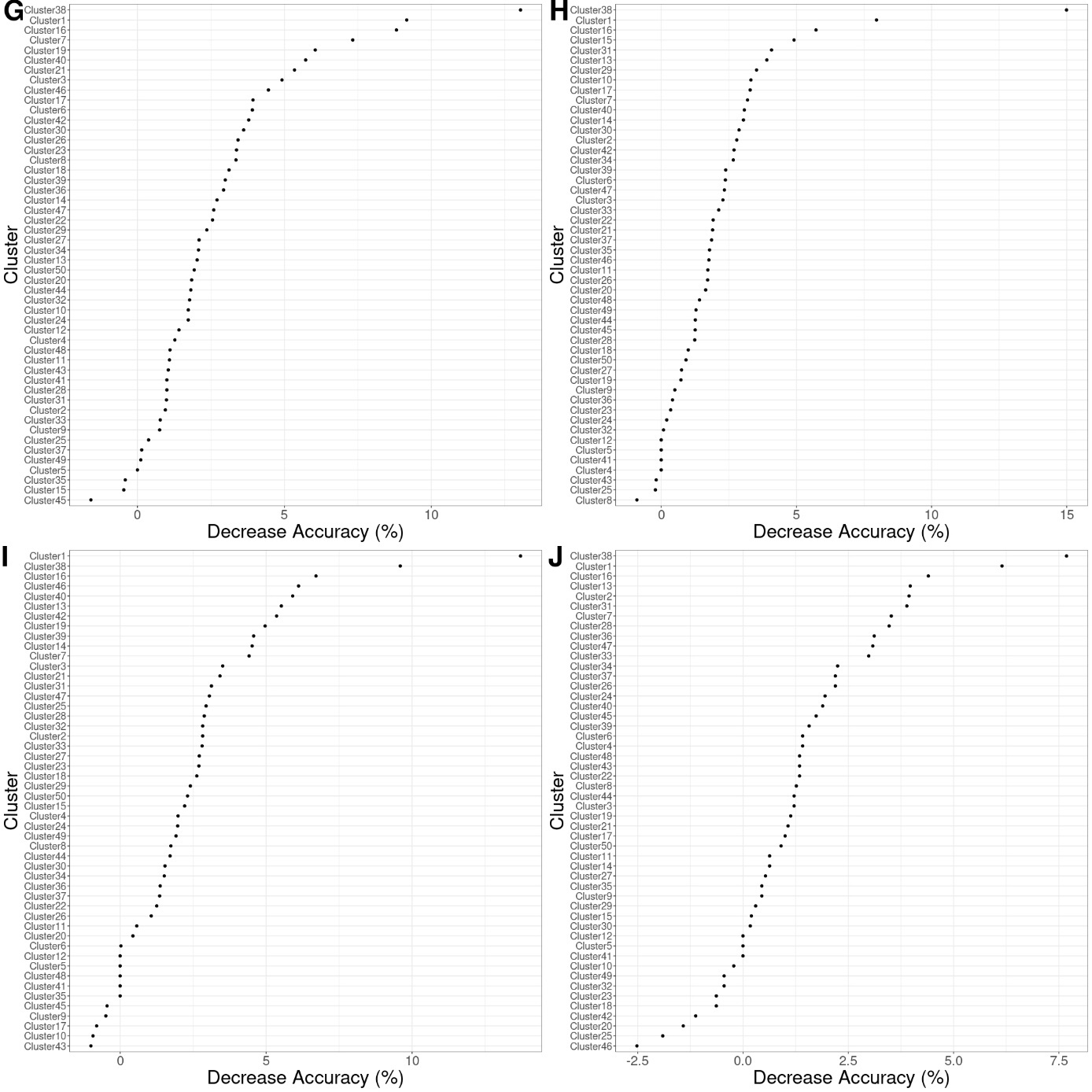


Figure S22. Variable importance per MOA class for *A. viscosus* after 24h of treatment. (A) ‘Control’ class, (B) ‘Cell Wall Synthesis’ class, (C) ‘DNA Replication’ class, (D) ‘DNA Transcription’ class, (E) ‘Folic Acid Metabolism’ class, (F) ‘Heat’ class, (G) ‘Membrane Disruption’ class, (H) ‘Protein Synthesis – 30S Inhibition’ class, (I) ‘Protein Synthesis – 50S Inhibition’ class, (J) ‘Protein Synthesis – tRNA Transcription’ class.


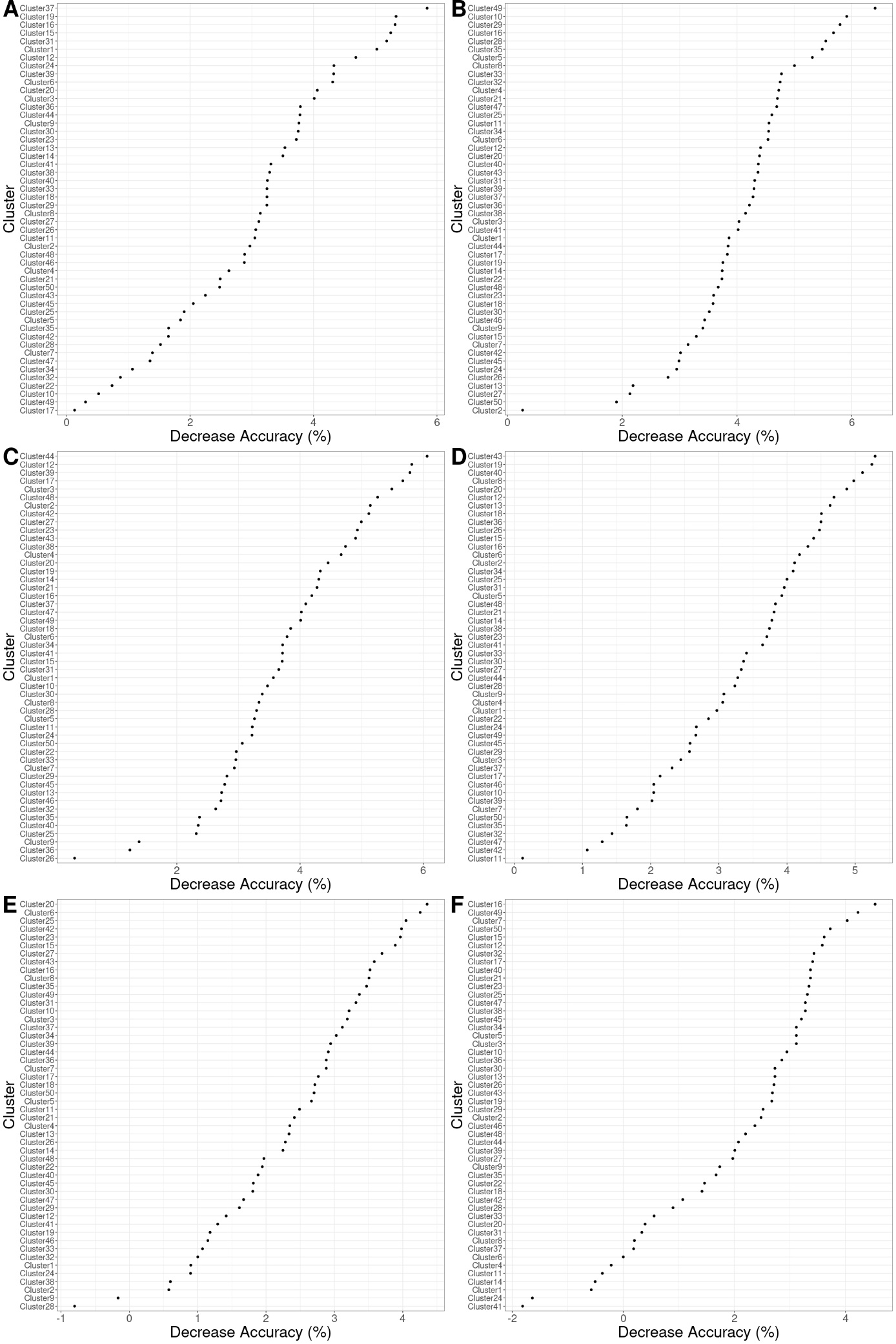


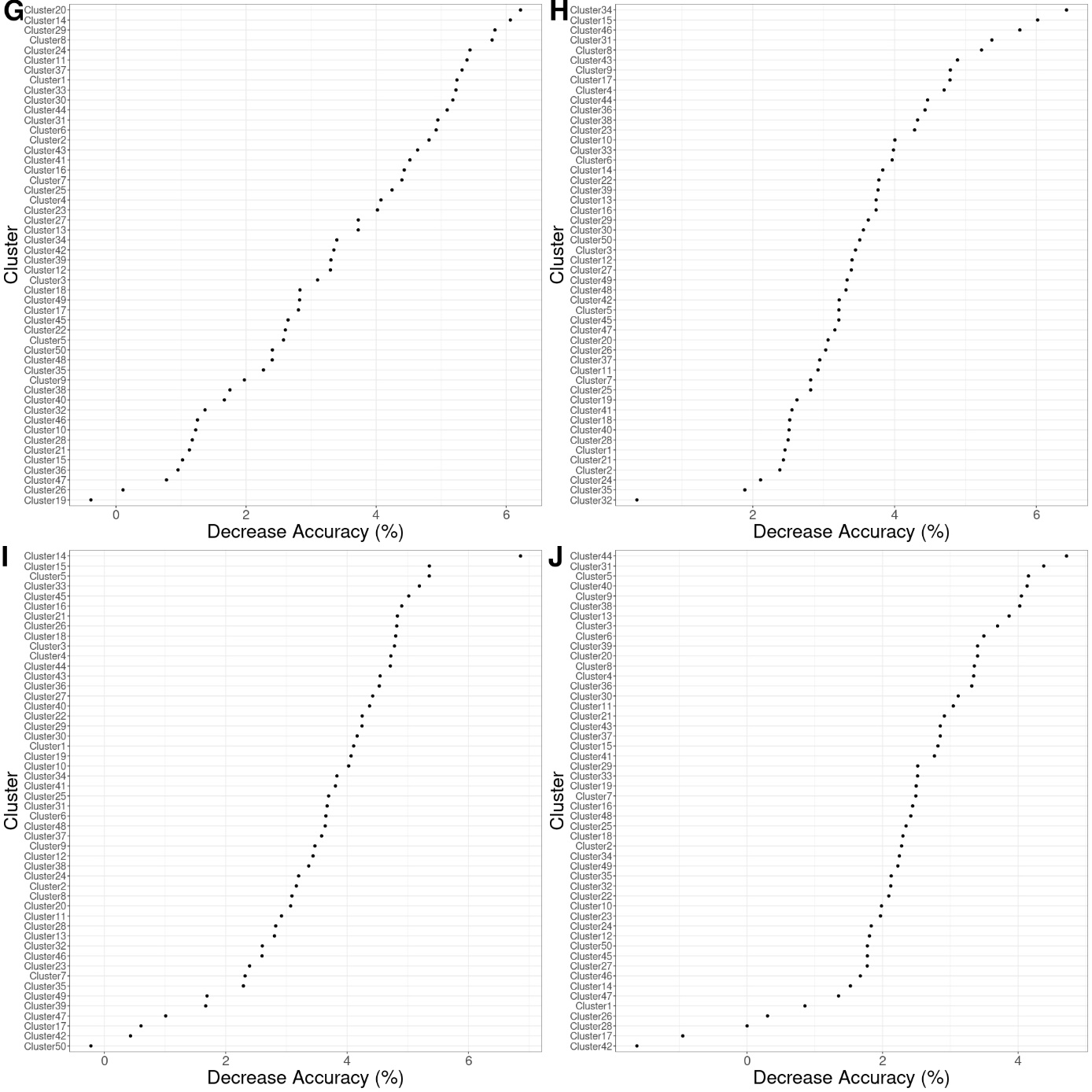


Figure S23. Variable importance per MOA class for *F. nucleatum* after 3.5h of treatment. (A) ‘Control’ class, (B) ‘Cell Wall Synthesis’ class, (C) ‘DNA Replication’ class, (D) ‘DNA Transcription’ class, (E) ‘Folic Acid Metabolism’ class, (F) ‘Heat’ class, (G) ‘Membrane Disruption’ class, (H) ‘Protein Synthesis – 30S Inhibition’ class, (I) ‘Protein Synthesis – 50S Inhibition’ class, (J) ‘Protein Synthesis – tRNA Transcription’ class.


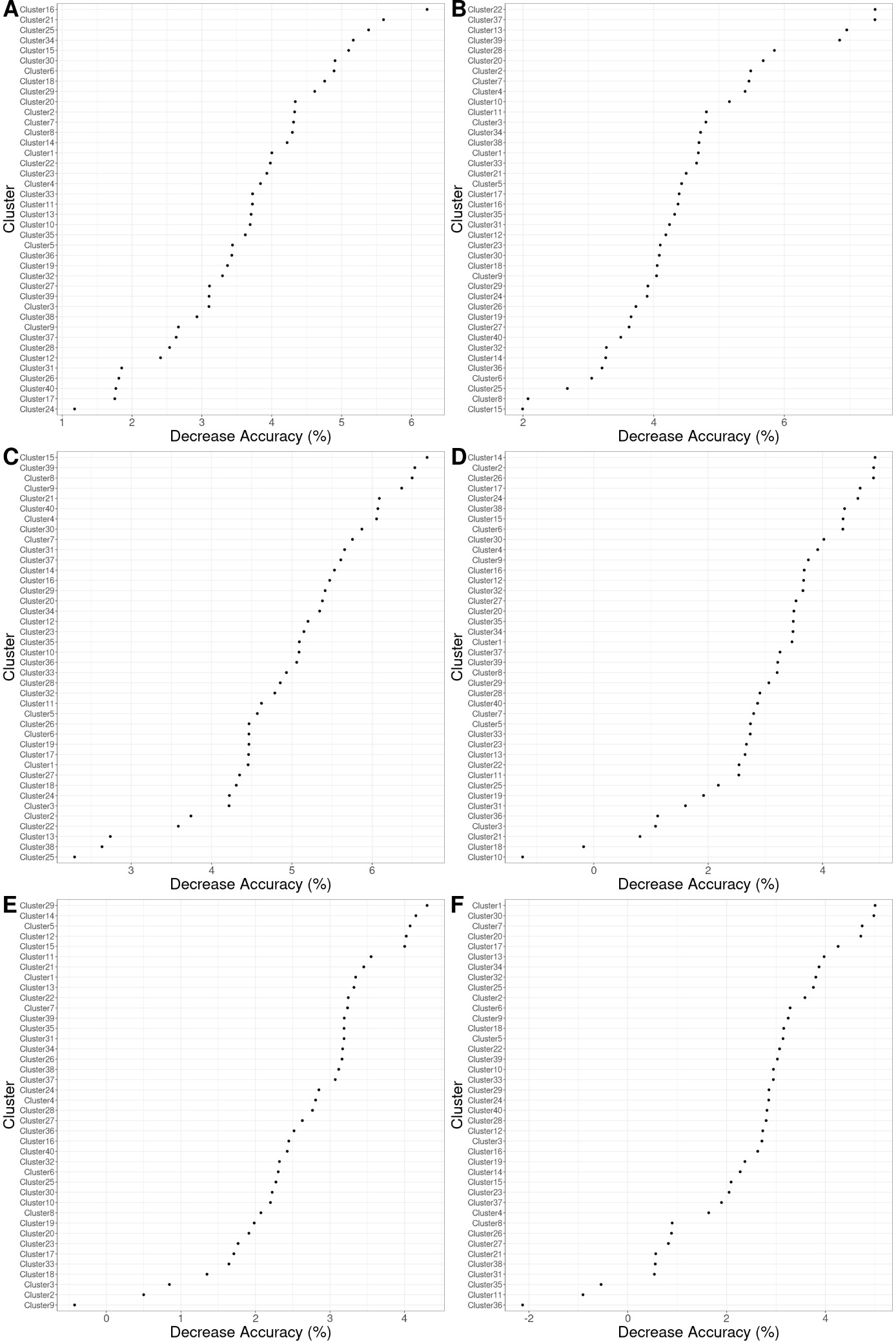


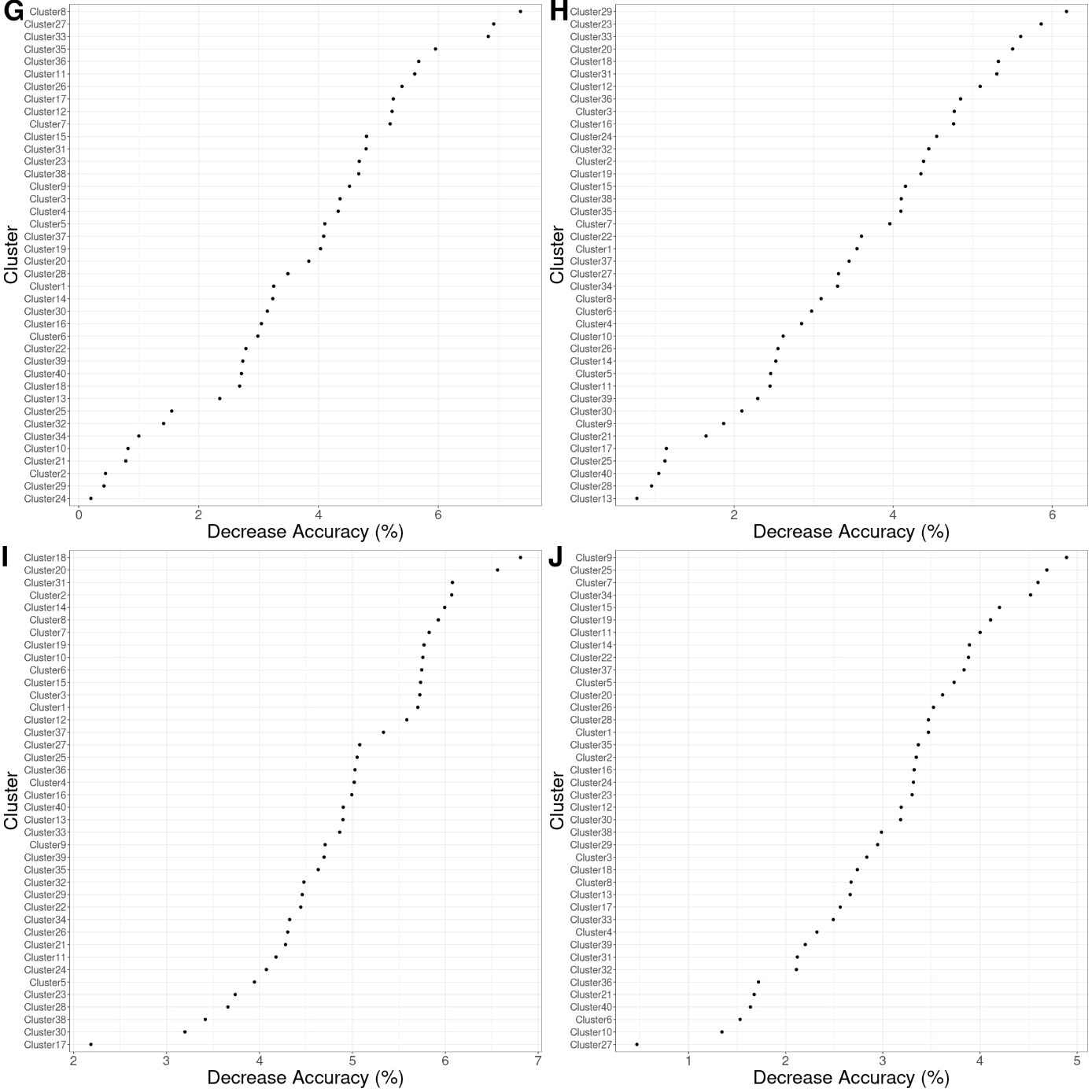


Figure S24. Variable importance per MOA class for *F. nucleatum* after 24h of treatment. (A) ‘Control’ class, (B) ‘Cell Wall Synthesis’ class, (C) ‘DNA Replication’ class, (D) ‘DNA Transcription’ class, (E) ‘Folic Acid Metabolism’ class, (F) ‘Heat’ class, (G) ‘Membrane Disruption’ class, (H) ‘Protein Synthesis – 30S Inhibition’ class, (I) ‘Protein Synthesis – 50S Inhibition’ class, (J) ‘Protein Synthesis – tRNA Transcription’ class.
